# Sirtuin Family and Diabetic Kidney Disease

**DOI:** 10.3389/fendo.2022.901066

**Published:** 2022-06-14

**Authors:** Che Bian, Huiwen Ren

**Affiliations:** ^1^ Department of Endocrinology and Metabolism, The Fourth Affiliated Hospital of China Medical University, Shenyang, China; ^2^ Advanced Institute for Medical Sciences, Dalian Medical University, Dalian, China

**Keywords:** Sirtuin (SIRT), diabetes, kidney, NAD+, diabetic kidney disease

## Abstract

Diabetes mellitus (DM) is gradually attacking the health and life of people all over the world. Diabetic kidney disease (DKD) is one of the most common chronic microvascular complications of DM, whose mechanism is complex and still lacks research. Sirtuin family is a class III histone deacetylase with highly conserved NAD^+^ binding domain and catalytic functional domain, while different N-terminal and C-terminal structures enable them to bind different deacetylated substrates to participate in the cellular NAD^+^ metabolism. The kidney is an organ rich in NAD^+^ and database exploration of literature shows that the Sirtuin family has different expression localization in renal, cellular, and subcellular structures. With the progress of modern technology, a variety of animal models and reagents for the Sirtuin family and DKD emerged. Machine learning in the literature shows that the Sirtuin family can regulate pathophysiological injury mainly in the glomerular filtration membrane, renal tubular absorption, and immune inflammation through various mechanisms such as epigenetics, multiple signaling pathways, and mitochondrial function. These mechanisms are the key nodes participating in DKD. Thus, it is of great significance for target therapy to study biological functions of the Sirtuin family and DKD regulation mechanism in-depth.

## 1 Introduction

Diabetes mellitus (DM) is a metabolic disease characterized by high blood glucose due to insulin secretion deficiency or biological function impairment (1). According to the International Diabetes Federation (IDF) data, the global adult DM population is over 537 million, 10.5% of the total population and shows a younger trend (2, 3), indicating that DM is gradually attacking the health and life of people all over the world. Therefore, studying the pathogenesis of complications in DM is still an urgent problem to be solved.

Diabetic kidney disease (DKD), also known as diabetic nephropathy (DN) (4) and is one of the most common chronic microvascular complications of DM with a 10%~40% DKD incidence in DM (5–7). The pathological changes are characterized by the continuous and slow development of proteinuria involving a complex pathological process of the glomerulus, renal tubules, microvessels, and other renal structures (8). It is of great significance to understand the pathogenesis of DKD and explore the targeting drugs related to DKD.

Silent information regulator 2-related enzymes (Sirtuin or SIRT) are the first discovered class III histone deacetylases (HDAC) of which NAD^+^-Sirtuin pathway is core in the energy metabolism for aging, cancer, cardiovascular, and cerebrovascular diseases as well as metabolic diseases (9, 10). Recent studies have shown that the Sirtuin family plays an important role in the biological mechanism and pathophysiological changes of DKD.

Thus, the aim of this review is to summarize the role of the Sirtuin family in DKD *via* displaying renal histological expressions, specific mechanisms, and renal injury of the Sirtuin family in different study models and reagents.

## 2 Pathophysiology of Diabetic Kidney Disease

**Figure 1:**
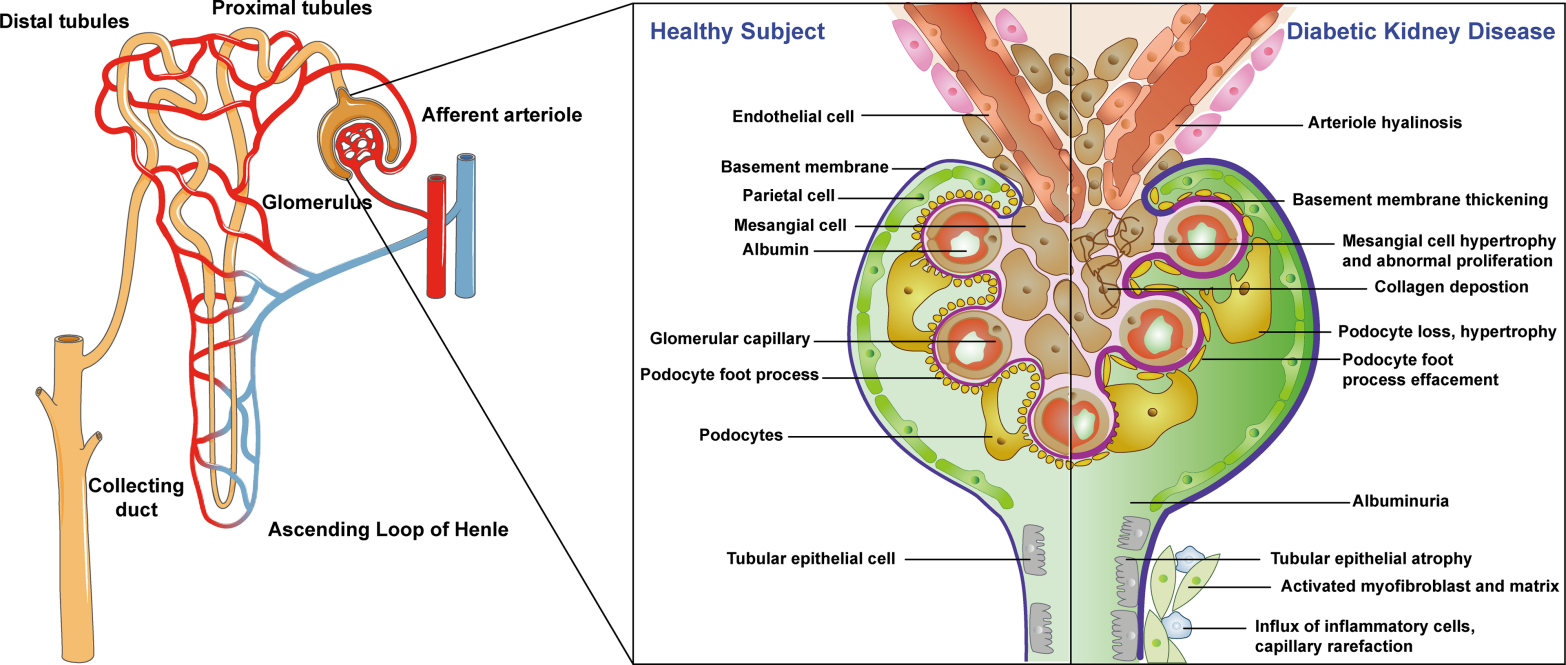
Physiological structure of nephron and pathologic process of diabetic kidney diseases. Left: whole kidney structure; middle: healthy nephron structure; right: the pathological changes of DKD.

The pathological changes of DKD start from basement membrane thickening in the early stage and gradually spread to the area of the glomerulus, microvessels, and renal tubules with characteristic changes of glomerular hyaline degeneration appearing in the late stage (11). Healthy nephron structures include endothelial cells, basement membrane, parietal cells, mesangial cells, glomerular capillaries, foot processes, podocytes, and renal tubular epithelial cells (12, 13). The pathological changes of DKD are mainly manifested as early glomerular basement membrane thickening, mesangial cell hypertrophy, abnormal hyperplasia, podocyte loss, hypertrophy, foot process disappearance with the later pathological manifestations of tubular epithelial atrophy, collagen deposition, activation of myofibroblasts and stroma, inflammatory cell influx, capillary thinning, and finally, arteriole hyaline degeneration (14–16). These pathological changes lead to persistent and slowly progressing proteinuria, eventually leading to end-stage renal disease (ESRD), which harms people’s health. The specific structure and pathological process are shown in **Figure 1**. The pathogenesis of DKD is a complex result of multiple factors with great significance to explore the pathogenesis of DKD for clinical diagnosis and treatment. Thus, it is of great significance to study the pathophysiological roles of DKD in different renal structures.

## 3 Sirtuin Family and Their Biological Functions

**Figure 2:**
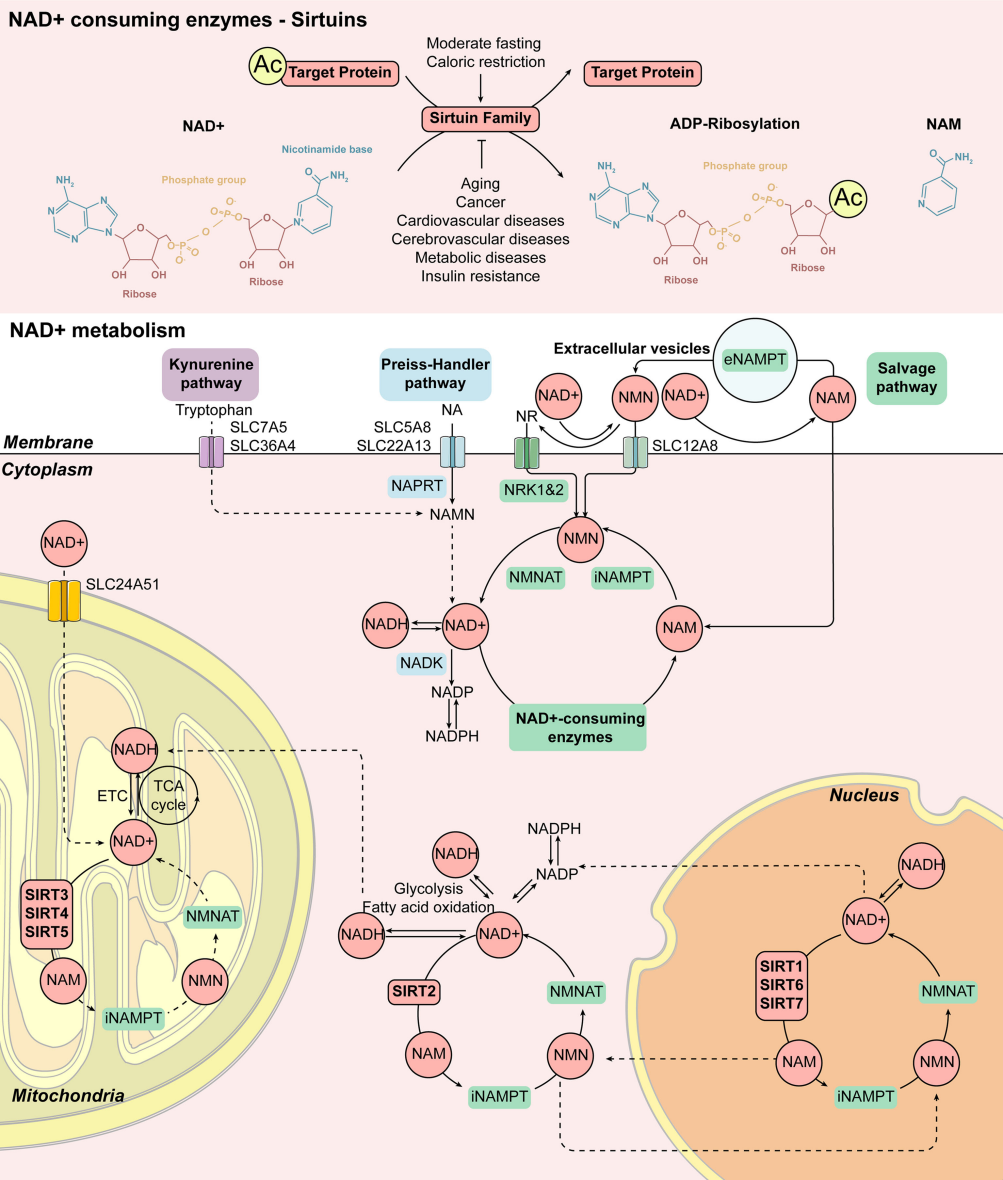
Cellular NAD^+^ metabolism induced by Sirtuin family. The enzymatic activity of the Sirtuin family is mainly to remove the acetyl group from the target protein. Firstly, NAD^+^ is cut into NAM and ADP-ribose, and the acetyl group on the target protein is transferred to ADP-ribose to form acetyl-ADP-ribose. Therefore, some members of the Sirtuin family can also play a role in ADP ribosyltransferase. The increase of NAD^+^ levels is closely related to the activation of the Sirtuin family members during moderate fasting and caloric restriction. On the contrary, aging, cancer, cardiovascular and cerebrovascular diseases as well as metabolic diseases such as insulin resistance lead to a decrease in NAD^+^ levels, which is related to the decrease in Sirtuin family activity. Mammalian cells can produce NAD^+^ from Tryptophan *via* the Kynurenine pathway or from NA, one of the forms of vitamin B3, *via* the Preiss-Handler pathway, while most NAD^+^ is recovered from NAM and NR *via* the Salvage pathway. NAD^+^ can be reduced to NADH during glycolysis, fatty acid oxidation, and the TCA cycle. NAD^+^ also acts as a substrate for enzymes such as Sirtuins, producing NAM as a byproduct, and affects metabolism, genomic stability, gene expression, inflammation, circadian rhythm, and stress resistance. This response pattern of the Sirtuin family is extensive. SIRT1, SIRT6, and SIRT7 exist in the nucleus, SIRT2 exists in the cytoplasm, while SIRT3, SIRT4, and SIRT5 exist in the mitochondrion. Abbreviations: Ac, acetylation; eNAMPT, extracellular nicotinamide phosphoribosyltransferase; ETC, electron transport chain; iNAMPT, intracellular nicotinamide phosphoribosyltransferase; MNAM, *N* ^1^-methylnicotinamide; NA, nicotinic acid; NAD^+^, nicotinamide adenine dinucleotide; NADH, nicotinamide adenine dinucleotide; NADK, NAD^+^ kinase; NADP/NADPH, nicotinamide adenine dinucleotide phosphate; NAM, nicotinamide; NAMN, nicotinamide mononucleotide; NAPRT, nicotinic acid phosphoribosyltransferase; NMN, nicotinamide mononucleotide; NMNAT, nicotinamide mononucleotide adenylyltransferases; NR, nicotinamide riboside; NRK1&2, nicotinamide riboside kinases 1 and 2; TCA, tricarboxylic acid.

Sirtuin family is the first discovered class III HDAC with the recognized family members SIRT1-7, which have different subcellular localizations. SIRT1, SIRT6, and SIRT7 are mainly distributed in the nucleus, while SIRT3, SIRT4, and SIRT5 are mainly located in the mitochondria and SIRT2 is mainly located in the cytoplasm (**Table 1** and **Figure 2**) (17). All of them have highly conserved nicotinamide adenine dinucleotide+ (NAD^+^) binding domain and catalytic functional domain, while different N-terminal and C-terminal structures enable them to bind different substrates (18). SIRT1-SIRT3 have strong deacetylase activity, SIRT4-SIRT7 are considered to be weak or even difficult to detect deacetylase activity and SIRT4 mainly has adenosine diphosphate (ADP)-ribosyltransferase activity (**Table 1**) (19, 20). Different enzymatic activities may be related to their different pathophysiological functions.

**Table 1:** The subcellular localization, substrates and enzyme function of Sirtuin family.

Sirtuin family member	Subcellular	Substrates	Enzyme function
	localization		
**SIRT1**	Nuclear, cytoplasmic	LKB1, p53, NFkB, PGC1α, HIF1α, HIF2α, CTIP2, Tat, p300, LXR, FXR, histone H1, histone H3, histone H4, eNOS, MEF2, Notch1, Ku70, WRN, NBS1, LKB1, hMOF, AceCS1, c-Myc, androgen receptor, cortactin, RARP1	Deacetylation Decrotonylase
**SIRT2**	Nuclear, cytoplasmic	LKB1, histone H3, histone H4, tubulin, p300, p65, PERCK1, FOXO1, FOXO3A, beta-secretase 1, p53, Par-3, CDK9, G6PD, PGAM, HIF1α, ALDH1A1, TUG, BubR1	Deacetylation
			Demyristoylase Decrotonylase
**SIRT3**	Mitochondrial	AceCS2, HMGCS2, ATP synthase F1, LCAD, SDH, Ku70, SOD2, FOXO3, aconitase 2, GDH, LKB1, MRPL10, LCAD, cyclophilin D, PDH, ALDH, Skp2, OGG1, Hsp10, GOT2, MDH	Deacetylation Decrotonylase
**SIRT4**	Mitochondrial	GDH, MCD, PDH, Hsp60, stress-70	Deacetylation
			ADP-ribosylation
			Lipoamidase
**SIRT5**	Mitochondrial	Cytochrome, CPS1, SOD1, urate oxidase, PML, VLCAD, Prx-1, HMGCS2, Hsp70, MCAD	Deacetylation
			Demalonylation
			Desuccinylation
			Deglutarylation
**SIRT6**	Nuclear	TNFα, histone H3, p70, Kup86, GCN5, KAP1, CtlP, Parp1, GEN1	Deacetylation
			ADP-ribosylation
**SIRT7**	Nuclear	Histone H3, PAF53, DNA-PK, GABPβ1, p53	Deacetylation

In mammals, NAD^+^ can be composed of four different biological precursors including two forms of niacin (NA), tryptophan, Vitamin B3, nicotinamide (NAM), and nicotinamide nucleoside (NR) (21) synthesized from the daily diet (including milk, meat, nuts, etc.) through three biological ways. The specific mechanism is shown in **Figure 2**. The enzymatic activity of the Sirtuin family is mainly deacetylase and ADP-ribosyltransferase to remove the acetyl group from the target protein with NAD^+^ transferred into NAM and acetyl-ADP-ribose (22). The main synthetic pathway of NAD^+^ is the Salvage pathway, in which NA, NAM, and NR, as the precursor, are converted to the intermediate nicotinamide mononucleotide (NMN), by the rate-limiting enzyme nicotinamide phosphoribose transferase (NAMPT). The intermediate, NMN can be converted into NAD^+^ by nicotinamide mononucleotide adenylate transferase (NMNAT). NAD^+^ produced by this pathway is consumed by a variety of enzymes including Sirtuins to regulate energy metabolism, mitochondrial function, and a variety of cellular responses to produce NAM and reuse it again *via* the Salvage pathway (23, 24). In the Preiss-Handler pathway, NA obtained from the daily diet can be transformed into mononucleotide nicotinate (NAMN) and nicotinamide adenine dinucleotide (NAAD) by key enzymes such as nicotinate phosphoribosyltransferase (NAPRT) and NMN adenylate transferase (NMNAT) and then further produce NAD^+^. In addition to these pathways, the *De Novo* pathway is another major source of NAD^+^ in the kidney. Tryptophan can be converted into NAMN by the quinoline phosphoribosyltransferase (QPRT) and then to NAD^+^ by the Preiss-Handler pathway. Supplementation of rate-regulating enzymes, precursors, and intermediates in these pathways could be used as potential treatments for metabolism-related diseases, including DM and obesity (9, 10, 25). Through the above-noted pathways, the Sirtuin family participates in the process of energy metabolism as deacetylase and ADP ribosyltransferase of NAD^+^.

Sirtuins active with the increase of NAD^+^ levels during moderate fasting and caloric restriction and decrease with the decrease of NAD^+^ levels for aging, cancer, cardiovascular and cerebrovascular diseases as well as metabolic diseases such as insulin resistance (9, 10, 25–30). Sirtuins mediate cell survival activation and other caloric restriction effects by regulating NAD^+^ enzymes, adenosine 5’-monophosphate (AMP)-activated protein kinase (AMPK), and mammalian target of rapamycin (mTOR) pathways (31, 32), and also promote oxidative phosphorylation, deacetylation of transcription factors, anti-inflammatory responses, and DNA repair, as well as inhibit glycolysis to combat oxidative stress (33, 34). Among them, SIRT1, as a star molecule in the family for its most wide studies, is involved in metabolism, immune response, aging regulation, and other various mechanisms. SIRT2 regulates cell division during DNA damage, SIRT3, SIRT4, and SIRT5 are the major regulators of mitochondrial energy metabolism and affect mitochondrial respiratory function *via* cytochrome C (CytC) (35), while SIRT6 and SIRT7 are strongly related to chromatin repair and transcription activation (36). The different biological functions of the Sirtuin family members may represent their different regulatory effects on DKD.

## 4 Localization of the Sirtuin Family in Kidney

Sirtuins only make action in the presence of coenzyme NAD^+^ in all living cells (23). Based on that, an increasing number of studies suggest that the maintenance of NAD^+^ levels and the corresponding decrease in Sirtuins activity can contribute to normal aging (37, 38). Expression levels of NAD^+^ may be closely related to the localization and expression of the Sirtuin family in renal tissues (21). According to existing studies, SIRT1 is widely expressed in renal tubular cells and podocytes (39), SIRT2 is mainly expressed in proximal epithelial renal tubular cells (40), SIRT3 has been described as a key regulator of mitochondrial dynamics in proximal epithelial tubular cells (41), little is known about the role of SIRT4 in the kidney (21), SIRT5 is highly expressed in proximal epithelial tubular cells (42), SIRT6 plays an important role of injury and fibrosis in podocytes and proximal epithelial tubular cells (43–45), while SIRT7 is expressed in proximal tubules and collecting tubules (46).

**Figure 3:**
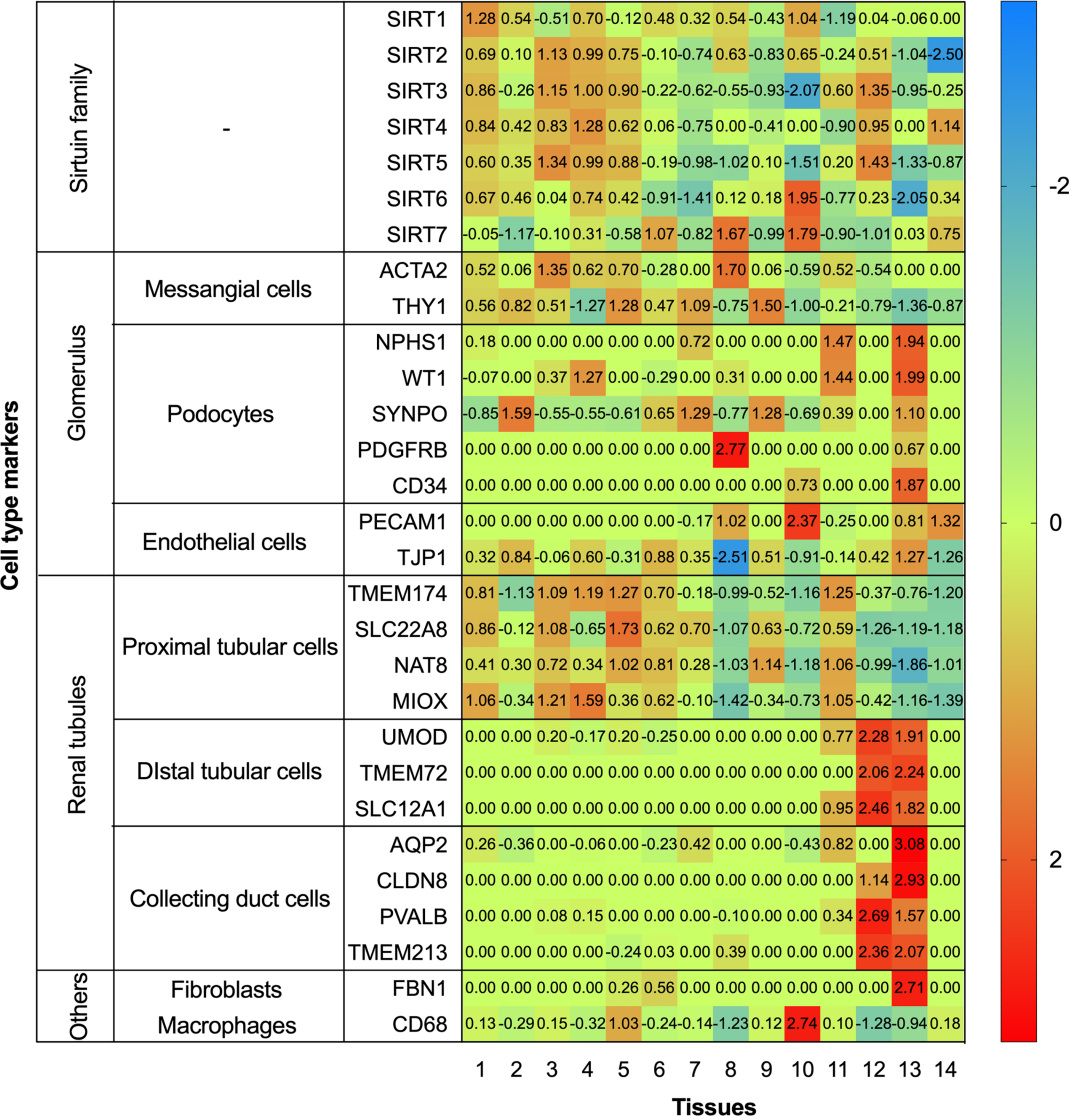
Heatmap of human Sirtuin family genes with different cell type markers. Single cell transcriptomics data for kidney tissues and peripheral blood mononuclear cells were analyzed. These datasets were respectively retrieved from the Single Cell Expression Atlas (https://www.ebi.ac.uk/gxa/sc/home), the Human Cell Atlas (https://www.humancellatlas.org/), the Gene Expression Omnibus (https://www.ncbi.nlm.nih.gov/geo/), the Allen Brain Map (https://portal.brain-map.org/), and the European Genome-phenome Archive (https://www.ebi.ac.uk/ega/).

**Figure 4:**
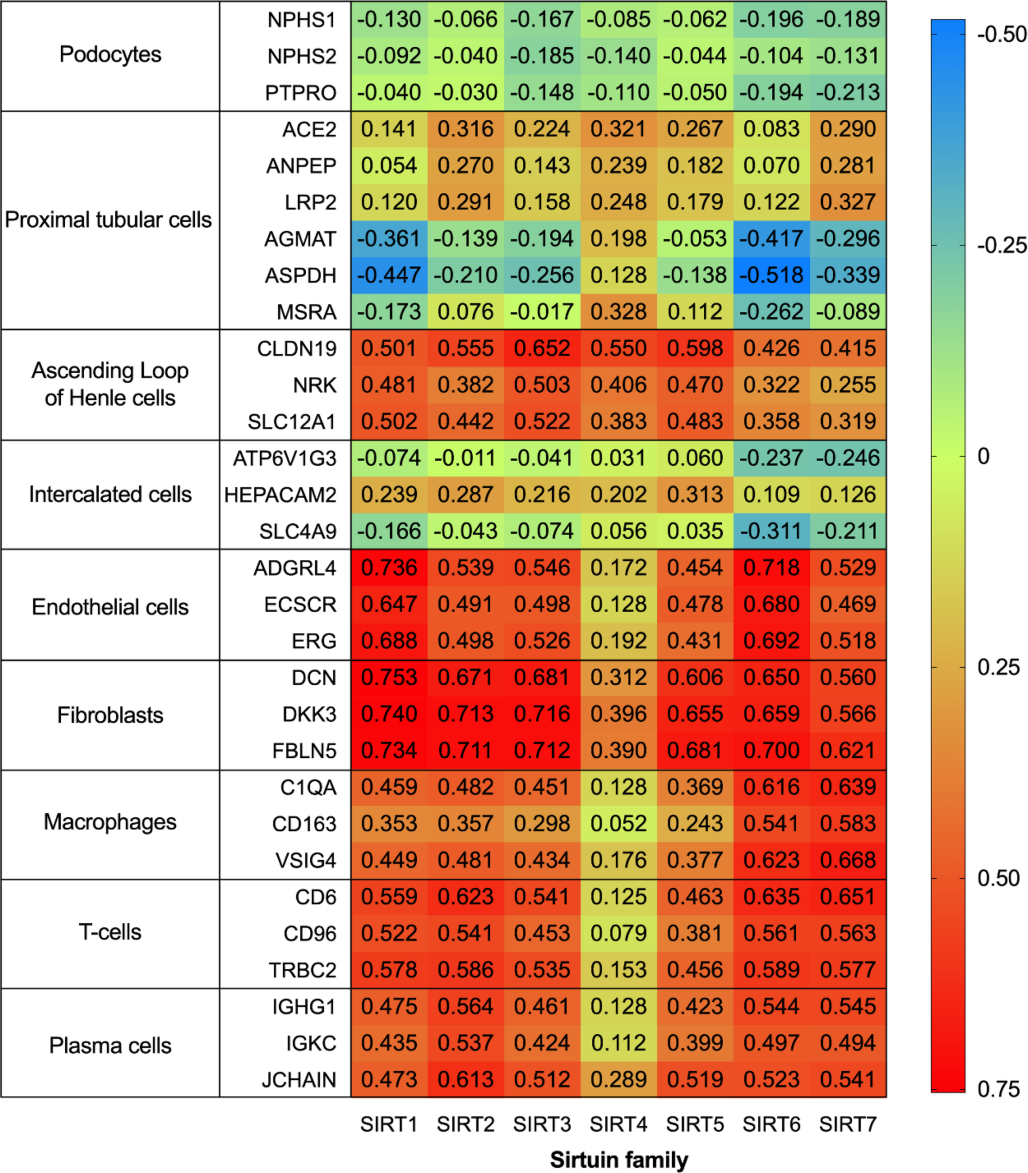
Correlation of human Sirtuin family transcriptome in renal tissues. The analysis was performed on data from RNA-seq of unfractionated tissue samples, which contained a mixed cell population. Across the respective sample sets the reference transcripts within each cell type panel correlated highly with each other, but not with those in the other panels. An integrative co-expression analysis was performed to determine the expression profile of each gene; genes highly correlated with all transcripts in only one reference panel were classified as enriched in that cell type.

To further systematically explore the localization of the Sirtuin family in kidneys, we conducted localization exploration through the Human Protein Atlas database. GraphPad Prism Version 8.0.0 for macOS Mojave 10.14.4 was used for both analyses with data collation and visual presentation (**Figures 3**, **4**). **Figure 3** indicated that SIRT1 had a similar expression with biomarkers in mesangial and proximal tubular cells, while SIRT2-5 showed a similar distribution similar to biomarkers in proximal tubular cells. SIRT6 and SIRT7 were similar to biomarkers in endothelial cells, while SIRT6 and biomarkers in proximal tubular cells also showed certain similarities. **Figure 4** showed that the whole family members had a low correlation to podocyte biomarkers in the filtration membrane system. In the renal tubules, SIRT4 had a higher correlation with proximal tubular cell biomarkers, while SIRT1 and SIRT6 had a lower correlation. The Sirtuin family expression was highly correlated in the thick segment of ascending limb. The high correlation of SIRT1-5 and low correlation of SIRT6-7 appeared in intercalated cells of the collecting duct and may be due to their different transporters and transport functions. Except for SIRT4, other family members were highly correlated with endothelial cells, fibroblasts, T cells, and macrophages, suggesting the involvement of renal interstitial fibrosis and inflammatory response. Furthermore, other members of the Sirtuin family, except SIRT4, are strongly correlated with plasma biomarkers. Our previous study found that serum SIRT6 decreased with different urinary albumin groups in patients with type 2 DM, which was correlated with urinary albumin excretion rate (47), also confirming their potential as biomarkers in circulation. This database research can partially supplement the defects of previous studies, however, it also has certain contradictions that need further exploration.

The expression of the Sirtuin family in kidneys shown in literature and databases is still contradictory. The reason may be due to the fact that most of Sirtuins’ studies have focused only on a single region of the kidney and have not conducted whole family comparisons at the same level. It is necessary to study the changes of the Sirtuin family in DKD by the spatiotemporal transcriptome sequencing in the whole renal tissues.

## 5 Role of the Sirtuin Family in Diabetic Kidney Disease

To explore the role of the Sirtuin family in DKD, previous studies have established different diabetes models, Sirtuin targeting animal models and a series of Sirtuin family targeting reagents. Studies have shown that the Sirtuin family regulates mesangial cell proliferation and hypertrophy, podocytes apoptosis, proximal tubular glucose metabolism, and renal tubular injury under DKD conditions and regulates podocytes mediated renal tubular, endothelial cells, and macrophages crosstalk *via* epigenetics of deacetylation and dephosphorylation, NAD^+^ involved mitochondrial function and multiple signaling pathway targets.

### 5.1 Study Models and Specific Reagents

Previous studies have established streptozotocin (STZ) induction, Otsuka-Long-Evans-Tokushima-Fatty (OLETF) rats, Akita mice, OVE26 mice, BTBR ob/ob mice, and endothelial nitric oxide synthase (eNOS)^-/-^ mice as different diabetes models and *Sirt1* global, podocyte-specific, and proximal tubule-specific overexpression and knockout mice as Sirtuin targeting animal models, and constructed a series of Sirtuin family targeting reagents represented by Resveratrol, and achieved some results as follows.

The mechanism of the Sirtuin family and DKD is still at the superficial stage and further mechanism exploration is needed. The research results of other pre-renal metabolic diseases (insulin resistance, obesity, non-alcoholic fatty liver disease, hyperuricemia, metabolic syndrome, etc.), renal injury diseases (acute kidney injury, chronic kidney diseases, or acute-chronic process), and post-renal obstructive diseases may have some enlightenment with the research on DKD. Moreover, the adverse pharmacokinetic and/or pharmacodynamic characteristics and uncertain effects may limit clinical application of Sirtuin reagents, which should be treated with caution. It is hoped that DKD therapeutic targets in different kidney tissues can be explored by combining different DKD and specific Sirtuin expressed animal models as well as reagents in the future.

#### 5.1.1 Diabetic Models

The most widely used model for DKD is STZ, which can enter islet cells through glucose transporter 2 (GLUT2) with a toxic effect on insulin, and induce insulin-secreting cells apoptosis. It is a typical model of type 1 or type 2 DM with or without a high-fat diet in rats and mice (48–50). It has a high modeling rate and mild pathological changes in DKD studies but with certain renal toxicity and multiple interventions with low doses. The effect of the Sirtuin family regulating STZ-induced DKD is mostly non-metabolic damage, mainly manifested as renal damage caused by mitochondrial dysfunction, oxidative stress, and inflammatory reaction, which may be associated with renal toxicity of STZ to some extent (**Table 2**). The expression of SIRT1, SIRT3, and SIRT6 were all decreased in the STZ-induced DKD model. SIRT1 mainly alleviated renal injury caused by mitochondrial oxidative stress and cellular inflammation through FoxO1, TGFβ1, and NFκB (58, 60, 69). SIRT3 maintained mitochondrial redox balance and regulated the glycolysis in renal tubular epithelial cells through PGC1α and TGFβ1 (101, 106). SIRT6 reduced proteinuria and damage in podocytes and mesangial cells by regulating H3K9ac and H3K56ac epigenetics, affecting nuclear translocation of FoxO1, and stabilizing inflammatory mediators (108, 110, 112, 113).

**Table 2:** Role of Sirtuin family in diabetic kidney diseases.

Sirtuin family	Animal model	Cell model	Sirtuin related Reagent	Molecular biology	Pathophysiology	References
**SIRT1**	—	SV40 MES13	—	Smad7 deacetylation	attenuate mesangial cell apoptosis	(51)
**SIRT1**	STZ-induced Sprague–Dawley rats	—	SIRT1 activator, resveratrol	change histone H3 phosphorylation, MAP kinase p38, SIR2 and p53 expression	—	(52)
**SIRT1**	—	HK-2	—	activate FoxO3a and catalase	release renal tubular cell apoptosis	(53)
**SIRT1**	db/db mice	mouse PTC	SIRT1 activator, resveratrol	regulate MnSOD activity	ameliorate oxidative stress in proximal tubules	(54)
**SIRT1**	db/db C57BLKS mice	mouse CIP	—	FoxO4 deacetylation	prevent podocyte apoptosis	(55)
**SIRT1**	diabetic Wistar fatty and lean rats	—	—	NFκB deacetylation	improve mitochondrial morphology and autophagosomes	(56)
**SIRT1**	aldosterone-induced mitochondrial dysfunction C57BL/6J mice	MPC5	SIRT1 activator, resveratrol	regulate PGC1α	reduce aldosterone-induced mitochondrial dysfunction and podocyte injury	(57)
**SIRT1**	STZ-induced Sprague-Dawley rats	—	SIRT1 activator, resveratrol	activate FoxO1	regulate oxidative stress and fibrosis	(58)
**SIRT1**	db/db C57BLKS/J mice	rat MC	SIRT1 activator, resveratrol	activate PGC1α, ERR1α, and SREBP1, decrease PI3K, Akt, FoxO3a	ameliorate glomerular matrix expansion and inflammation	(59)
**SIRT1**	STZ-induced Sprague-Dawley rats	rat MC	SIRT1 activator, resveratrol	activate Nrf2/ARE, reduce fibronectin and TGFβ1, increase HO1	reduce mesangial cell oxidative stress	(60)
**SIRT1**	kidney- and proximal tubules-specific *Sirt1* knockout, STZ-, FK866-, 5/6 Nephrectomy-induced nephrectomized and db/db mice	mouse CIP, HK-2	—	epigenetically suppress Claudin1	participate in crosstalk between podocytes and renal tubules: SIRT1 in proximal tubules protects against albuminuria by maintaining NMN around glomerulus, thus influencing podocyte function	(61)
**SIRT1**	STZ-induced Sprague-Dawley rats	mouse CIP, mouse GEC	SIRT1 activator, resveratrol	down-regulate VEGF and VEGFR2	regulate angiogenesis in podocyte and endothelial cells	(62)
**SIRT1**	db/db, podocyte-specific *Sirt1* knockout mice	human CIP	—	NFκB and STAT3 deacetylation	attenuate proteinuria and podocyte injury	(63)
**SIRT1**	STZ-induced Sprague–Dawley rats	—	—	increase HO1, loss FoxO1	suppress oxidative stress and extracellular matrix deposition	(64)
**SIRT1**	STZ-induced diabetic spontaneously hypertensive rats	human MC	—	decrease NOX4 and TGFβ1, maintaining PARP1, intracellular NAD^+^/NADH ratio, AMP/ATP ratio, Smad3 deacetylation	ameliorate mesangial cell extracellular matrix accumulation	(65)
**SIRT1**	STZ-induced, *Sirt1* transgenic C57BL/6J mice	HEK293A	—	regulate p300, ET1 and TGFβ1	protect from renal injury	(66)
**SIRT1**	—	rat MC	SIRT1 activator, resveratrol	inhibit HIF1α	inhibit mesangial cell inflammation and fibrosis	(67)
**SIRT1**	db/db C57BLKS/J mice	human GEC	SIRT1 activator, resveratrol	decrease FoxO1, FoxO3a, and SREBP1, increase PPARγ, PGC1α, ERR1α, and pACC	ameliorate lipotoxicity, oxidative stress, apoptosis and endothelial cell dysfunction	(68)
**SIRT1**	STZ-induced Wistar rats	—	SIRT1 activator, resveratrol	normalize TGFβ1, fibronectin, NFκB, Nrf2, and FoxO1	protect renal oxidative damage	(69)
**SIRT1**	STZ-induced Wistar rats	HK-2	SIRT1 activator, resveratrol	p53 deacetylation	ameliorate renal tubular injury	(70)
**SIRT1**	STZ-induced Wistar albino rats	—	—	inhibit NFκB	alleviate renal oxidative stress	(71)
**SIRT1**	STZ-induced eNOS^−/−^ mice	mouse CIP	—	down-regulate NOX4, increase NFκB deacetylation	attenuates podocytes injury	(72)
**SIRT1**	OVE26 mice, podocyte-specific *Sirt1* overexpression mice	human CIP	SIRT1 agonist, BF175	activate PGC1α	attenuate podocyte loss and glomerular oxidative stress	(73)
**SIRT1**	db/db C57BL/KsJ mice	mouse MC	—	regulate HIF1α	alleviate mesangial cell proliferation and renal fibrosis	(74)
**SIRT1**	db/db C57BL/6 mice	LLC-PK1 porcine renal epithelial cells	—	up-regulate GLUT2, down-regulate SGLT2	high basolateral glucose in renal tubules increases SGLT2 and decreases SIRT1 and GLUT2	(75)
**SIRT1**	—	HK-2	—	regulate LC3II, ATG5 and ATG7	regulate autophagy and fibrosis in renal proximal tubules	(76)
**SIRT1**	OLETF rats	HK-2, HEK293T	—	regulate TGFβ1	attenuate EMT and proximal tubule cell fibrosis	(77)
**SIRT1**	STZ-induced diabetic CD-1 mice	mouse CIP	SIRT1 activator, resveratrol	regulate PGC1α, increased MnSOD, inhibit ROS	attenuation of mitochondrial oxidative stress, inhibit podocyte and renal tubular epithelial cell apoptosis	(78)
**SIRT1**	STZ-induced Sprague–Dawley rats	—	—	up-regulate Nrf2/HO1	renal tubules dysfunction and oxidative stress	(79)
**SIRT1**	STZ-induced C57BL/6J mice	—	—	regulate PGC1α	improve kidney fibrosis and mitochondrial biogenesis	(80)
**SIRT1**	—	HEK293	—	down-regulate phosphorylate mTOR	prevent kidney cell damage	(81)
**SIRT1**	STZ-induced C57BL/6J mice with HFD	—	—	activate AMPK/PGC1α	improve renal fibrosis, inflammation, and oxidative stress	(82)
**SIRT1**	STZ-induced C57BL/6 mice	human CIP, rat GEC, rat MC	—	PGC1α and FoxO1 deacetylation	balance mitochondrial dysfunction, biogenesis, and mitophagy, regulate podocyte injury and proteinuria	(83)
**SIRT1**	STZ-induced Sprague–Dawley rats	rat MC	—	regulate FoxO1	alleviate abnormal mesangial cells proliferation	(84)
**SIRT1**	STZ-induced C57BL/6 mice	mouse MC	—	regulate PGC1α, Nrf1, mtTFA, mtDNA copy, and ATP	affect mitochondrial biogenesis and function in mesangial cells	(85)
**SIRT1**	STZ-induced Sprague–Dawley rats with HFD	—	SIRT1 inhibitor, EX527	regulate FoxO1	alleviate oxidative stress and structural changes of glomerulus, inhibit extracellular matrix	(86)
**SIRT1**	STZ-induced CD1 mice, db/db C57BLKS/J mice	human CIP	SIRT1 activator, resveratrol; SIRT1 inhibitor, EX527	phosphorylation SIRT1 S47 to S47A decrease ROS and cytochrome c release, increase ATP	regulate podocyte mitochondrial function	(87)
**SIRT1**	STZ- induced C57BL/6J mice with HFD	mouse CIP	—	inhibit NFκB	inhibit podocyte oxide stress and inflammation	(88)
**SIRT1**	STZ-induced Sprague–Dawley rats	—	—	inhibit NLRP3, IL1β, TNFα and NFκB	regulate renal oxidant-antioxidant balance, dampen inflammation, attenuate collagen accumulation	(89)
**SIRT1**	STZ-induced Sprague–Dawley rats with HFD	mouse CIP	—	activate phosphorylate AMPK and inhibit phosphorylate NFκB	block podocyte oxidative stress and inflammatory responses	(90)
**SIRT1**	STZ induced Wistar rats	—	—	inhibit phosphorylate FoxO3a, Claudin1	suppress renal oxidative stress	(91)
**SIRT1**	db/db C57BL/6J mice	MPC5, rat MC, GEC, HK-2, NRK-52E, RAW 264.7	—	activate AMPK-SREBP1	participate in podocyte lipid metabolism	(92)
**SIRT1**	STZ-induced C57BL/6 mice	HK-2	SIRT1 inhibitor, EX-527	induce NFκB and STAT3 dephosphorylation and deacetylation	reduce tubular epithelial cell oxidative stress, apoptosis, inflammation response, and EMT	(93)
**SIRT1**	db/db C57BLKs/J mice	SV40 MES13	SIRT1 inhibitor, EX527	compete with PARP1 for NAD^+^, activate AMPK/PGC1α	ameliorate mesangial cell extracellular matrix accumulation	(94)
**SIRT1**	STZ-induced C57BL/6 mice with HFD	—	—	upregulate PGC1α	upregulate in diabetic mice kidney	(95)
**SIRT2**	caloric restriction C57BL/6 mice	HEK293, HEK293T	—	FoxO3a deacetylation, increase FoxO DNA binding, Kip1, MnSOD, and Bim	oxidative stress increases SIRT2 in kidney cells	(96)
**SIRT1 and SIRT3**	—	rat MC	—	maintaining intracellular NAD^+^/NADH ratio, blocked Akt, augmented AMPK, prevent mTOR	inhibit mesangial cell hypertrophy	(97)
**SIRT1 and SIRT3**	Zucker Diabetic Fatty Rats with HFD	—	SIRT1 inhibitor, EX527	regulate Claudin1	revealed expansion of the extracellular mesangial matrix and suppression of glomerulosclerosis	(98)
**SIRT3**	—	HK-2	—	regulate Akt/FoxO1 and FoxO3a activity	antagonize tubular epithelial cell apoptosis	(99)
**SIRT3**	Zucker Lean Rats and Zucker Diabetic Fatty Rats	HK-2	—	IDH2 deacetylation, decrease SOD2, CD38, increase NAD^+^/NADH ratio	decrease tubular cell damage, mitochondrial oxidative stress and morphologic alterations	(100)
**SIRT3**	STZ-induced CD-1 and C57Bl6 KsJ mice, Akita mice	HK-2	—	inhibit TGFβ1/Smad3, HIF1α, and PKM2 dimer formation	abnormal glycolysis and EMT in tubular epithelial cells	(101)
**SIRT3**	—	HK-2	—	increase phosphorylated Akt and FoxO3a	protect tubular epithelial cells against oxidative stress and apoptosis	(102)
**SIRT3**	db/db C57BL/6J mice	mouse PTC	SIRT3 inhibitor, 3-TYP	inhibit BNIP3	ameliorates oxidative stress and cell apoptosis in proximal tubular cells	(103)
**SIRT3**	BTBR ob/ob mice	—	—	activate SOD2, restore PGC1α	attenuate albuminuria, ameliorate glomerular damage, reduce podocyte injury, tubule-glomerulus retrograde interplay	(104)
**SIRT3**	Zucker Lean Rats and Zucker Diabetic Fatty Rats	HK-2	—	restore intracellular NAD +/NADH ratio	reduce tubulointerstitial fibrosis and tubular cell damage	(105)
**SIRT3**	STZ-induced Wistar rats with HFD	—	—	activate PGC1α and SOD2	maintaining mitochondrial redox equilibrium	(106)
**SIRT4**	—	mouse CIP	—	down-regulate NOX1, Bax and phosphorylated p38, up-regulate Bcl2, attenuate TNFα, IL1β and IL6	inhibit podocyte apoptosis	(107)
**SIRT6**	podocyte-specific *Sirt6* knockout, STZ-induced diabetic, adriamycin-induced nephropathy, db/db C57BL/6 mice	rat MC, rat GEC, HK-2, human CIP	—	histone H3K9 deacetylation, inhibit Notch1 and Notch4 transcription	exacerbate podocyte injury and proteinuria	(44)
**SIRT6**	STZ-induced C57BL/6 mice	human CIP	—	increase H3K9ac and H3K56ac	suppress mitochondrial dysfunction and apoptosis in podocytes	(108)
**SIRT6**	STZ-induced C57BL/6J mice	HK-2	—	regulate TIMP1	regulate tubular basement membrane thickening, collagen deposition, and albuminuria	(109)
**SIRT6**	STZ-induced diabetic rats	THP-1, MPC5	—	upregulate Bcl2 and CD206, decrease Bax and CD86	activate M2 macrophages regulating immune response, protect podocyte injury	(110)
**SIRT6**	db/db mice	HK-2	—	Smad3 deacetylation	regulate tubular injury and renal function loss	(111)
**SIRT6**	STZ-induced Kunming mice	Rat MC	—	regulate IL6, IL1β, TNFα and MPO	regulate proliferation, migration, fibrosis and inflammatory response in mesangial cells	(112)
**SIRT6**	STZ-induced diabetic rats	mouse PTC	—	affect nuclear translocation of FoxO1	reverse the glucose reabsorption and gluconeogenesis effect	(113)

GEC, Glomerular endothelial cells; VSMC, vascular smooth muscle cells; CIP, conditionally immortalized podocytes; MC, mesangial cells; PTC, proximal tubular cells. Specific cell lines: HUVEC, human umbilical vein endothelial cells; MPC5, mouse podocyte cells; SV40 MES13, mouse mesangial cell line; NRK-52E, rat renal tubular epithelial cells; HK-2, human tubular epithelial cells; HEK293, HEK293A, HEK293T, human embryonic kidney cells; THP-1, human peripheral blood monocyte; RAW 264.7, mouse macrophage-like cell line. Special treatment: streptozotocin (STZ), high-fat diet (HFD), OLETF rats, OVE26 mice, db/db mice, BTBR ob/ob mice and Akita mice were seen in the main body of text.

Another model commonly used to study the Sirtuin family is db/db mice, which are homozygous mutant mice of leptin receptor gene encoded by db genes, with spontaneous hyperglycemia and insulin resistance, and the occurrence of DKD complications at 8-12 weeks and general life of about 10 months. The db/db mice are widely used in obesity and type 2 DM research and are ideal models for studying early DKD lesions. The Sirtuin family’s regulation of db/db mice induced DKD model is mostly metabolic changes, mainly manifested as pre-renal glucolipid metabolism disorders caused by glucotoxicity and lipid peroxidation (shown in **Table 2**). SIRT1, SIRT3, and SIRT6 were all decreased in this model consistent with STZ-induced DKD. However, SIRT1 can improve glycolipid toxicity and oxidative stress *via* SREBP1 and GLUT2, thus reducing proteinuria and improving kidney injury (59, 68, 75, 92). SIRT3 reduced oxidative stress and apoptosis of renal tubules by inhibiting BNIP3 (103). SIRT6 regulated renal tubular damage and protected renal function through Smad3 deacetylation (111). In addition, there are no studies on histones regulation by the Sirtuin family in db/db mice, which is worthy of further exploration.

Only a few other models have focused on OLETF rats, Akita mice, OVE26 mice, BTBR ob/ob mice, and eNOS^-/-^ mice. OLETF rats are animals of high appetence induced by loss of rat cholecystokinin A receptor genes. It is similar to the DKD model of human type 2 DM with mild pathological changes, which can better study the whole duration of DKD (114). Studies have shown that inhibition of miRNAs can reduce EMT and renal fibrosis in HK-2 cells of diabetic OLETF rats, and SIRT1 is identified as the target of these two miRNAs (77), speculating that SIRT1 has a regulatory effect on OLETF rates DKD model. Akita mice are islets β apoptosis model affecting the normal folding of insulin protein induced by dominant missense mutations of Ins2 genes with the A7 position of insulin changed from cysteine to tyrosine. Its renal lesions are mild and survival is difficult with better usage of early DKD filtration membrane study (115, 116). The study on SIRT3 mentioned Akita mice with DKD development (117), but there was no specific study on Sirtuin family members (101). OVE26 mice are FVB line early-onset type 1 diabetic mice with calmodulin overexpression in islet β cells leading to islet defect. Mice have the characteristics of DKD at 4-6 months, with the irreversible disappearance of podocytes in the later stage and high mortality (118). Studies have shown that both *Sirt1* overexpression and exogenous activation reduced podocyte loss and oxidative stress in OVE26 mice (73). BTBR has the characteristics of natural insulin resistance, while ob/ob mice leptin mutant genes lead to loss of satiety and spontaneous obesity in mice. The combination of the two models can establish the DKD model from early to late but with a high price (119, 120). Studies have shown that SIRT3 decreased in BTBR ob/ob mice renal tissues and SIRT3 reduced proteinuria improved glomerular and podocyte damage and had retrograde changes in renal tubular and glomerular (104). The eNOS^-/-^ mice model accelerates renal injury through vascular endothelial dysfunction and hypertension. Its combination with STZ and db/db mice is an ideal model for studying the late stage of DKD but it is difficult for feeding (121, 122). Puerarin treatment increased SIRT1 mRNA and protein in podocytes and significantly alleviated albuminuria and diabetic kidney injury in diabetic eNOS^-/-^ mice (72), thus speculating the relationship between SIRT1 and eNOS^-/-^ mice. These studies of the Sirtuin family are only preliminary and further exploration of pathological changes in different stages of DKD caused by different models is still needed.

#### 5.1.2 Sirtuin Gene Editing Models

The Sirtuin-specific model constructed by gene-editing technology is a supplement to DKD model research (**Table 2**). *Sirt1* overexpression in transgenic mice attenuated ET-1, TGF-β1, microalbuminuria, glucose-induced cell damage markers, and fibronectin in diabetic renal tissues (66). Podocyte-specific overexpression attenuated the progression of diabetic glomerulopathy and proteinuria (73). Proximal tubule-specific *Sirt1* transgenic and knockout mice suggested the occurrence of diabetic glomerular pathological prevention and aggravation, while non-diabetic knockout mice showed proteinuria, suggesting that SIRT1 in proximal tubules affected glomerular function (61). *Sirt1* podocyte-specific knockout db/db mice had more proteinuria, renal injury, and acetylation of p65 and STAT3 (63). *Sirt6* podocyte-specific knockout intensified podocyte damage and proteinuria (44).

#### 5.1.3 Sirtuin Reagents

In addition, newly developed Sirtuin-related activators and antagonists also have certain applications in DKD research. The SIRT1 activator, Resveratrol, has been widely used in DKD studies (**Table 2**). *In vivo* administration of SIRT1 inhibitor, EX-527, for 10 weeks significantly reduced blood glucose and kidney weight in high-fat diet-induced Zucker rats, decreased blood urea nitrogen, serum creatinine, microalbumin, urine excretion, and inhibited the histopathological expansion of the extracellular mesangial matrix and glomerulosclerosis (98). Mechanically, EX-527 regulated the accumulation of extracellular matrix in mesangial cells *via* the AMPK-PGC1α pathway (94) eliminated the protective effect of Na_2_S_4_ in DKD renal tubular cells (93) and inhibited autophagy (86). SIRT1 agonist, BF175, increased PGC1α activation and protected podocyte mitochondria injury induced by high glucose. *In vivo* BF175 treatment for 6 weeks significantly reduced albuminuria and glomerular injury (73). SIRT3 inhibitor, 3-TYP, inhibited mitochondrial function, apoptosis, and reactive oxygen species (ROS) production in proximal tubular cells under high glucose conditions (103). The research and development, as well as the DKD application of these gene-editing model animals and reagents, play an effective role in the study of the Sirtuin family in DKD.

Sirtuin family targeting reagents, especially Resveratrol, are considered to have high potential in clinical molecular targeted therapy for DKD (123). It is found that its antioxidant and anti-inflammatory properties are associated with diabetes, obesity, cardiovascular diseases, and cancer in related clinical trials (124), however, the adverse pharmacokinetic and/or pharmacodynamic characteristics, such as poor bioavailability, may limit its wide clinical application (125), and even some studies have shown no significant effect on renal function (126). Therefore, these results should be treated with caution before the clinical transformation.

### 5.2 Renal Injury

Early changes of DKD are focused on the glomerular filtration membrane, while renal tubules and other renal areas for the later changes. The Sirtuin family has been widely studied for early filtration membrane injury, including mesangial matrix thickening, abnormal mesangial cell proliferation, and podocytes damage. NAD^+^ is concentrated in renal tubules, thus, Sirtuin is also closely related to renal tubular injury as well as crosstalk between podocytes and other renal cells.

#### 5.2.1 Glomerular Filtration Membrane

Early changes of DKD are mesangial matrix thickening, abnormal proliferation of mesangial cells, and podocytes changes. The cause of continuous urinary protein changes is mainly focused on abnormal changes in the filtration membrane (11). SIRT1 decreased in renal tubules and glomerulus of diabetic nephropathy patients (127, 128). SIRT6 was detected to decrease in renal biopsy samples of patients with diseases in podocytes including DKD and its expression was correlated with glomerular filtration rates (44). Studies have shown that high glucose reduced SIRT1, SIRT3, SIRT4, and SIRT6 levels in podocytes (83), suggesting that the Sirtuin family can regulate DKD filtration membrane changes.

Abnormal changes in the DKD filtration membrane mainly focus on the structural and functional changes of mesangial cells in the early stage. Our previous studies explored the protective effect of SIRT1 deacetylase modification on DKD mesangial cell injury (84, 129). SIRT1 regulated hypertrophy and proliferation of mesangial cells (74, 84), mesangial matrix deposition (59, 65, 94), oxidative stress (60, 73) and fibrosis (67, 74) in diabetic model. SIRT1 also regulated mesangial cell matrix deposition (97) and mesangial hypertrophy (98) together with SIRT3. Additionally, SIRT6 had an effect on the proliferation, migration, fibrosis, and inflammation in mesangial cells (112).

**Figure 5:**
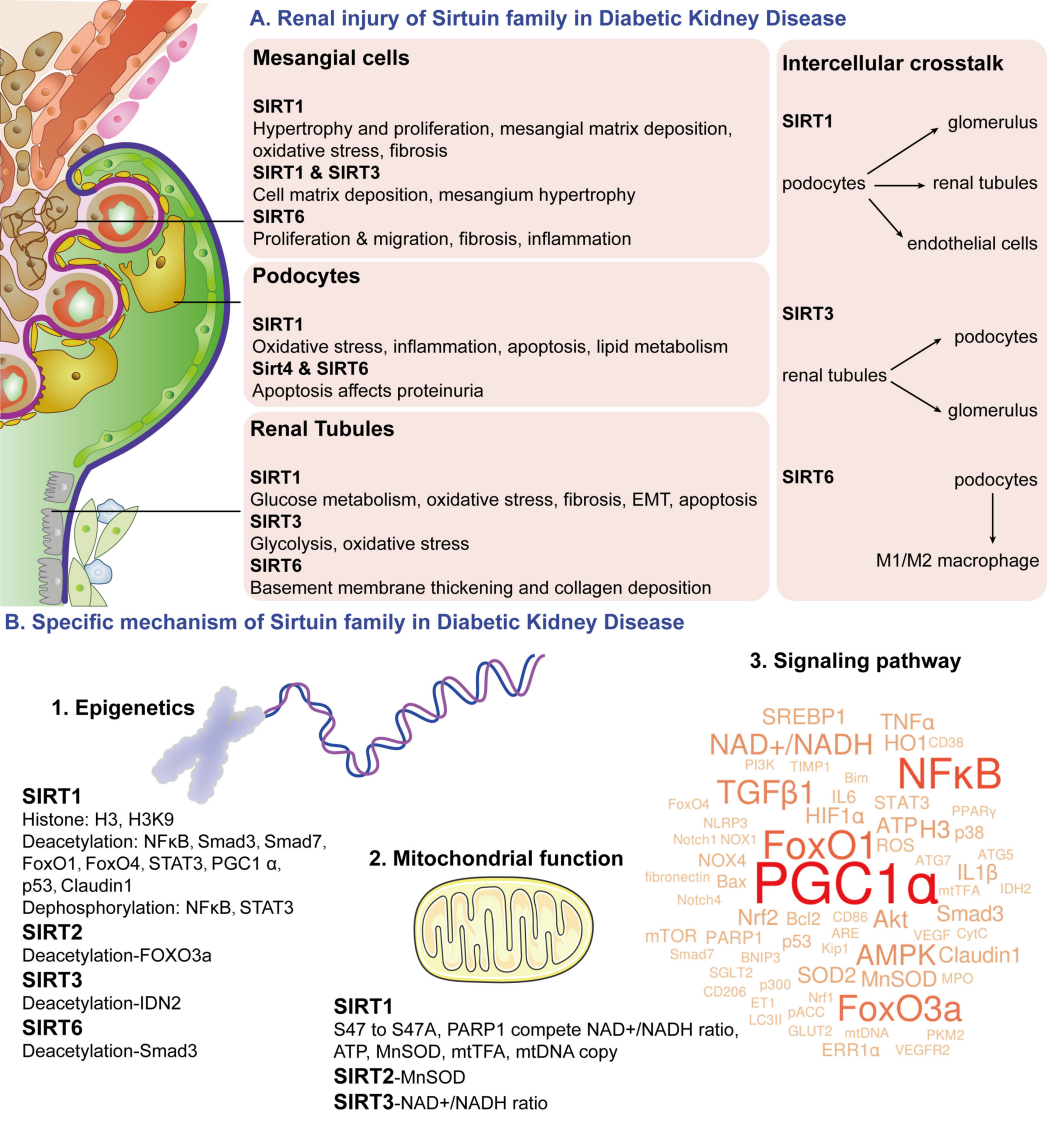
Schematic diagram of Sirtuin family. The Sirtuin family is a deacetylase with an NAD^+^ binding domain that consumes NAD^+^ to regulate energy metabolism. Sirtuin family regulates mesangial cell proliferation and hypertrophy, podocytes apoptosis, glucose metabolism in proximal tubules, and renal tubular injury in DKD pathophysiological changes through epigenetics of acetylation and dephosphorylation, NAD^+^ induced mitochondrial function, and multiple signaling pathway targets. It also participates in podocytes mediated renal tubular cells, endothelial cells, and macrophages crosstalk. **(A)** renal injury of Sirtuin family in DKD; **(B)** specific mechanism of Sirtuin family in DKD.

Major changes in the late stage of DKD are the filtration membrane changes caused by podocytes. Among them, SIRT1 mainly regulated oxidative stress and inflammation in podocytes under high glucose conditions (88, 90) and then slowed down the apoptosis process (55, 78). SIRT1 was also involved in the lipid metabolism of podocytes (92). In addition, SIRT4 and SIRT6 affected proteinuria production by regulating podocyte apoptosis (44, 107, 108). Studying the changes in the filtration membrane is helpful to prove the mechanism of the Sirtuin family regulating proteinuria production through the DKD filtration membrane system (**Figure 5**).

#### 5.2.2 Renal Tubules

NAD^+^ metabolism is enriched in the proximal tubular area (21), which may be closely related to the localization and expression of the Sirtuin family in renal tissues. Therefore, the pathophysiological role of renal tubules in DKD should not be ignored (130). The main function of diabetic renal tubules is glucose reabsorption and metabolism. Sodium-glucose cotransporter 2 (SGLT2) transports glucose from proximal tubular lumen into proximal tubular cells through active transport in the apical membrane (131, 132). Then glucose easily diffuses into the blood along the concentration gradient through glucose transporter 1/2 (GLUT1/2) after reaching the basement membrane to complete the glucose reabsorption process (133). It was found that high glucose increased SGLT2 in the basement membrane of renal tubules and decreased SIRT1 and GLUT2 (75). SIRT3 in diabetic kidneys was inhibited, showing fibrotic reprogramming related to abnormal renal glycolysis (101). SIRT6 reversed the glucose reabsorption and gluconeogenesis effect (113). These Sirtuin family changes in renal glucose metabolism are of great significance for renal blood glucose regulation.

Except for renal glucose metabolism, late renal changes of DKD mainly include renal tubular epithelial atrophy, collagen deposition, activation of myofibroblast and matrix, inflammatory cell influx, and epithelial-mesenchymal transition (EMT) (134, 135). Studies showed that SIRT1 mainly regulated cell apoptosis induced by renal tubular oxidative stress (54, 78, 79, 93), fibrosis (76, 77) and EMT (77, 93). SIRT3 was mainly related to renal tubular oxidative stress (100, 102, 103). SIRT6 affected basement membrane thickening in renal tubules and collagen deposition (109). These above-noted studies all indicate that the Sirtuin family regulates late DKD pathological changes of oxidative stress, fibrosis, and EMT in renal tubules (**Figure 5**).

#### 5.2.3 Intercellular Crosstalk

More interestingly, the Sirtuin family is closely related to the regulation of podocytes-mediated renal cell crosstalk in DKD studies. It was found that SIRT1 reduced mitochondrial oxidative stress in DKD renal tissues and inhibited cell apoptosis in podocytes and tubular epithelial cells (78). Proximal tubular SIRT1 affects podocyte function by maintaining periglomerular NMN concentration (61). The protective effect of SIRT3 on DKD proteinuria and glomerular changes may be due to retrograde tubule-glomerular interaction. Upregulation of SIRT3 and NAMPT in renal tubules can provide NMN required by diabetic podocytes and other glomerular cells and ultimately provide glomerular NAD^+^ to further increase SIRT3 activity, forming a virtuous cycle (104). The Sirtuin family interaction in podocyte-tubules provides NAD^+^ as energy for various regions of renal tissues and participates in maintaining normal cell metabolism. SIRT1 in podocytes and endothelial cells was also linked, which regulated both podocytes and endothelial angiogenesis (62). In addition, the Sirtuin family also regulated podocyte-macrophage crosstalk. High glucose promoted M1 macrophages transformation, podocyte apoptosis, and decreased SIRT6. SIRT6-overexpressed macrophages could transform into M2 macrophages and protect podocytes from high glucose damage (110). It suggests that podocytes-macrophages crosstalk of the Sirtuin family provides a theoretical basis for protecting against DKD injury (**Figure 5**).

### 5.3 Specific Mechanism

Sirtuin is the first discovered class III HDAC with different epigenetic enzyme effects in DKD. Intracellular NAD^+^/NADH ratios maintain the activity of the Sirtuin family for mitochondrial biogenesis. Multiple Sirtuin-related targets found through literature learning and bioinformatics are of great significance to explore the pathogenesis of DKD.

#### 5.3.1 Epigenetics

Epigenetics mainly involves DNA methylation, histone modification, and chromosomal remodeling, among which histone covalent modification includes methylation, acetylation, phosphorylation, and ubiquitination (136–138). Acetylation modifications mainly include “Reader” for specific recognition of protein lysine, “Writer” as acetyltransferase, and “Eraser” as deacetylation HDACs (139, 140), while Sirtuin is the first discovered class III HDAC. In DKD studies, SIRT1 was involved in the phosphorylation of histone H3 (52) and the acetylation of H3K9 (44, 108). Moreover, SIRT1 has a variety of deacetylase effects. Sirtuin studies on DKD has reported target proteins including Nuclear factor kappa B (NFκB) (56, 63, 72, 93), Smad3 (65), Smad7 (51), Forkhead Box Protein O1 (FoxO1) (83), Forkhead Box Protein O4 (FoxO4) (55), signal transducer and activator of transcription 3 (STAT3) (63, 93), Peroxsome proliferator-activated receptor-γ coactlvator-1α (PGC1α) (83), p53 (70), and Claudin1 (61). Meanwhile, SIRT1 could make actions for dephosphorylating NFκB and STAT3 (93). In studies of other Sirtuin family members with DKD models, SIRT2 can deacetylate Forkhead Box Protein O3a (FoxO3a) (96), SIRT3 can deacetylate isocitrate dehydrogenase 2 (IDH2) (100), and SIRT6 can deacetylate Smad3 (111). Polydeacetylation and dephosphorylation targets researches of the Sirtuin family are of great significance for DKD function regulation (**Figure 5**).

#### 5.3.2 Mitochondrial Function

The Sirtuin family participates in various deacetylation for mitochondrial biogenesis, oxidative stress, inflammatory cell apoptosis, and autophagy through cellular NAD^+^ usage. The imbalance of NAD^+^ and NADH is a marker of DM and its chronic complications (141). In the diabetic state, the glycolysis pathway and tricarboxylic acid cycle are activated, NAD^+^ is reduced to NADH, leads to NADH overload and ROS increase, and further leads to oxidative stress. NAD^+^ decrease also results in acetylation of proteins such as PGC1α involved in oxidative stress and mitochondrial biogenesis, ultimately leading to DKD progression (142, 143).

In the mitochondrial function changes, poly ADP-ribose polymerase 1 (PARP1) is a DNA repair and protein modification enzyme that competes with NAD^+^ to cause mitochondrial dysfunction (144). Manganese superoxide dismutase (MnSOD) is a key antioxidant enzyme in mitochondria (145). Mitochondrial transcription factor A (mtTFA) is a key regulator for mitochondrial DNA (mtDNA) transcription and replication, maintaining the normal function of mitochondria and preventing mitochondrial damage (146). It was found that NAD^+^ maintained intracellular NAD^+^/NADH ratio as well as SIRT1 and SIRT3 activities (65, 97). SIRT1 phosphorylation mutations from S47 to S47A can regulate podocyte mitochondrial function by reducing ROS and CytC release and increasing ATP (87). SIRT1 can compete with poly ADP-ribose polymerase 1 (PARP1) for NAD^+^ (94) and maintain PARP1, intracellular NAD^+^/NADH ratio, AMP/ATP ratio (65), MnSOD activity (54), mtTFA and mtDNA copy number (85). SIRT2 can increase MnSOD activity (96), while SIRT3 can restore intracellular NAD^+^/NADH ratios (100, 105). In conclusion, SIRT1-3 reduces oxidative stress by improving energy metabolism and plays an important role in regulating mitochondrial function in DKD (**Figure 5**).

#### 5.3.3 Signaling Pathway

Moreover, we conducted machine learning for all the literature on the Sirtuin family and the wordle of the Sirtuin family signaling pathway in DKD was visualized (**Figure 5**). The wordcounts with the top highest frequency were PGC1α, NFκB, FoxO1, FoxO3a, transforming growth factor-β1 (TGFβ1), and AMPK, suggesting a close relationship of Sirtuin family and these pathway factors.

PGC1α plays a role in energy metabolism processes including adaptive thermogenesis, mitochondrial biosynthesis, liver glycogenesis, and fatty acid β oxidation. Therefore, changes of PGC1α occur the most in the DKD process of the Sirtuin family, which is closely related to mitochondrial oxidative stress and energy metabolism (57, 68, 73, 78, 80, 82, 83, 85, 94, 104, 106). PGC1α can regulate the non-ligand-dependent orphan nuclear receptor, estrogen-related receptor α (ERRα), and regulation of the Sirtuin family in DKD is also accompanied by changes of ERRα (59, 68). Meanwhile, the regulation of PGC1α by the Sirtuin family is also accompanied by the changes of FoxO transcription factors (59, 68, 83) due to the same mitochondrial energy regulation.

NFκB is involved in the immune and inflammatory response to external stimuli. It mainly plays a role in mitochondrial oxidative stress in DKD regulation of Sirtuin family (56, 69, 71, 72, 88–90) and STAT3-mediated inflammatory regulation (63, 88–90, 93).

FoxO transcription factor family plays an important role in aging and longevity. They mainly involved in the regulation of Sirtuin family in the oxidative stress response in various regions of DKD kidney (53, 58, 64, 68, 69, 86, 91, 96, 102). Our previous studies have found that SIRT1 alleviated oxidative stress and enhanced autophagy in renal tissues of diabetic rats by regulating FoxO1 phosphorylation, which also confirms the main role of Sirtuin (84). The Akt signaling pathway can regulate the changes of the FoxO transcription factor, thus the regulation of SIRT1/3-FoxOs in DKD always involves the Akt participation (59, 99, 102). In addition, changes of PGC1α often happen in the regulation of Sirtuin-FoxOs in DKD, which may be related to their common energy regulation function (59, 68, 83).

TGFβ1 is a key molecule of renal fibrosis. SIRT1 induces glomerular extracellular matrix proliferation and changes of Collagen type IV (ET-1), Collagen 1α, and non-collagenous glycoprotein, Fibronectin, *via* TGFβ1/Smad pathway in early DKD (65, 66, 69, 77). Endogenous antioxidant stress results in renal fibrosis and changes of nuclear factor erythroid 2-related factor 2 (Nrf2)- antioxidant responsive element (ARE) pathway (60, 69). Our previous research also indicates that SIRT1 regulates DKD oxidative stress and fibrosis in diabetic rats through HIF1α and TGFβ1/Smad3 signaling pathway (129). Additionally, SIRT3 in the DKD model was associated with TGFβ1-mediated renal fibrosis (101). Therefore, regulation of the Sirtuin family in DKD *via* TGFβ1 is mainly related to extracellular matrix accumulation at the early stage leading to renal fibrosis.

AMPK is a key molecule in the regulation of biological energy metabolism. Our previous research found that SIRT6 regulated sterol regulatory element-binding protein 1c mediated glucolipid metabolism in the liver and pancreas through AMPKα-mTORC1 (147). In the DKD study, SIRT1 regulated podocyte fatty acid synthesis *via* AMPK-SREBP1 (92), podocyte inflammation by AMPK-NFκB (90), glucolipid metabolism, and mitochondrial function by AMPK-PGC1α (82), fibronectin in mesangial matrix deposition (94), and protein synthesis and mesangial cell hypertrophy through AMPK-mTOR (97). In addition, we found that SIRT1 activated by metformin could regulate DKD progression in mesangial cells (84). All the above suggests that SIRT1-AMPK induced glucolipid metabolism of the Sirtuin family plays a key role in DKD mesangial cells and podocytes.

## 6 Conclusion

The Sirtuin family is a deacetylase with NAD^+^ binding domains. SIRT1-7, as members of the Sirtuin family, have different subcellular localization and catalytic enzyme activities, which consume NAD^+^ to regulate energy metabolism, mitochondrial function, redox, and other cellular reactions. Studies have shown that the Sirtuin family regulates mesangial cell proliferation and hypertrophy, podocytes apoptosis, proximal tubular glucose metabolism, and renal tubular injury under DKD conditions. Meanwhile, the Sirtuin family is closely related to the regulation of podocytes mediated renal tubular, endothelial cells, and macrophages crosstalk. These pathophysiological changes are regulated by epigenetics of deacetylation and dephosphorylation, NAD^+^ involved mitochondrial function changes and multiple signaling pathway targets. However, literature and databases still show contradictories in the renal expression of the Sirtuin family, which needs further exploration. The mechanism of the Sirtuin family and DKD is still at the superficial stage. The research results of other pre-renal metabolic diseases, renal injury diseases, and post-renal obstructive diseases may have some enlightenment with the research on DKD. With the rapid development of modern science and technology, different gene-specific expression animals and DKD models, as well as reagents, have been discovered. The Sirtuin family is expected to become an important therapeutic target of DKD by regulating different regions of renal tissues.

## Author Contributions

CB: searched data, drafted and revised the manuscript. HR: inspiration, edited and critically revised the manuscript for intellectual contents. All authors contributed to the article and approved the submitted version.

## Funding

This study was supported by the Doctoral Research Initiation Fund Project of Liaoning Province (Grant No. 2021-BS-206) and Shenyang Young and Middle-aged Innovation Support Program (RC210460).

## Conflict of Interest

The authors declare that the research was conducted in the absence of any commercial or financial relationships that could be construed as a potential conflict of interest.

## Publisher’s Note

All claims expressed in this article are solely those of the authors and do not necessarily represent those of their affiliated organizations, or those of the publisher, the editors and the reviewers. Any product that may be evaluated in this article, or claim that may be made by its manufacturer, is not guaranteed or endorsed by the publisher.

## References

[1] American Diabetes Association. 2. Classification and Diagnosis of Diabetes: Standards of Medical Care in Diabetes-2021. Diabetes Care (2021) 44:S15–33. doi: 10.2337/dc21-S002 33298413

[2] American Diabetes Association Professional Practice Committee. 2. Classification and Diagnosis of Diabetes: Standards of Medical Care in Diabetes-2022. Diabetes Care (2022) 45:S17–S38. doi: 10.2337/dc22-S002 34964875

[3] Sun H Saeedi P Karuranga S Pinkepank M Ogurtsova K Duncan BB. IDF Diabetes Atlas: Global, Regional and Country-Level Diabetes Prevalence Estimates for 2021 and Projections for 2045. Diabetes Res Clin Pract (2022) 183:109119. doi: 10.1016/j.diabres.2021.109119 34879977 PMC11057359

[4] KDOQI. KDOQI Clinical Practice Guidelines and Clinical Practice Recommendations for Diabetes and Chronic Kidney Disease. Am J Kidney Dis (2007) 49:S12–154. doi: 10.1053/j.ajkd.2006.12.005 17276798

[5] Nelson RG Newman JM Knowler WC Sievers ML Kunzelman CL Pettitt DJ. Incidence of End-Stage Renal Disease in Type 2 (Non-Insulin-Dependent) Diabetes Mellitus in Pima Indians. Diabetologia (1988) 31:730–6. doi: 10.1007/BF00274774 3240833

[6] Rosolowsky ET Skupien J Smiles AM Niewczas M Roshan B Stanton R. Risk for ESRD in Type 1 Diabetes Remains High Despite Renoprotection. J Am Soc Nephrol (2011) 22:545–53. doi: 10.1681/ASN.2010040354 PMC3060448 21355053

[7] Alicic RZ Rooney MT Tuttle KR. Diabetic Kidney Disease: Challenges, Progress, and Possibilities. Clin J Am Soc Nephrol (2017) 12:2032–45. doi: 10.2215/CJN.11491116 PMC5718284 28522654

[8] Stevens PE Levin A Kidney Disease: Improving Global Outcomes Chronic Kidney Disease Guideline Development Work Group Members. Evaluation and Management of Chronic Kidney Disease: Synopsis of the Kidney Disease: Improving Global Outcomes 2012 Clinical Practice Guideline. Ann Intern Med (2013) 158:825–30. doi: 10.7326/0003-4819-158-11-201306040-00007 23732715

[9] Covarrubias AJ Perrone R Grozio A Verdin E. NAD+ Metabolism and its Roles in Cellular Processes During Ageing. Nat Rev Mol Cell Biol (2021) 22:119–41. doi: 10.1038/s41580-020-00313-x PMC7963035 33353981

[10] Xu J Kitada M Koya D. NAD+ Homeostasis in Diabetic Kidney Disease. Front Med (Lausanne) (2021) 8:703076. doi: 10.3389/fmed.2021.703076 34368195 PMC8333862

[11] Marshall CB. Rethinking Glomerular Basement Membrane Thickening in Diabetic Nephropathy: Adaptive or Pathogenic? Am J Physiol Renal Physiol (2016) 311:F831–43. doi: 10.1152/ajprenal.00313.2016 PMC6121820 27582102

[12] Ndisang JF. Glomerular Endothelium and its Impact on Glomerular Filtration Barrier in Diabetes: Are the Gaps Still Illusive? Curr Med Chem (2018) 25:1525–9. doi: 10.2174/0929867324666170705124647 28685678

[13] Tang C Livingston MJ Liu Z Dong Z. Autophagy in Kidney Homeostasis and Disease. Nat Rev Nephrol (2020) 16:489–508. doi: 10.1038/s41581-020-0309-2 32704047 PMC7868042

[14] Fu H Liu S Bastacky SI Wang X Tian X-J Zhou D. Diabetic Kidney Diseases Revisited: A New Perspective for a New Era. Mol Metab (2019) 30:250–63. doi: 10.1016/j.molmet.2019.10.005 PMC6838932 31767176

[15] Chen S-J Lv L-L Liu B-C Tang R-N. Crosstalk Between Tubular Epithelial Cells and Glomerular Endothelial Cells in Diabetic Kidney Disease. Cell Prolif (2020) 53:e12763. doi: 10.1111/cpr.12763 31925859 PMC7106959

[16] Zhang Y Jin D Kang X Zhou R Sun Y Lian F. Signaling Pathways Involved in Diabetic Renal Fibrosis. Front Cell Dev Biol (2021) 9:696542. doi: 10.3389/fcell.2021.696542 34327204 PMC8314387

[17] O’Callaghan C Vassilopoulos A. Sirtuins at the Crossroads of Stemness, Aging, and Cancer. Aging Cell (2017) 16:1208–18. doi: 10.1111/acel.12685 PMC5676072 28994177

[18] Poulose N Raju R. Sirtuin Regulation in Aging and Injury. Biochim Biophys Acta (2015) 1852:2442–55. doi: 10.1016/j.bbadis.2015.08.017 PMC4682052 26303641

[19] Haigis MC Guarente LP. Mammalian Sirtuins–Emerging Roles in Physiology, Aging, and Calorie Restriction. Genes Dev (2006) 20:2913–21. doi: 10.1101/gad.1467506 17079682

[20] Finkel T Deng C-X Mostoslavsky R. Recent Progress in the Biology and Physiology of Sirtuins. Nature (2009) 460:587–91. doi: 10.1038/nature08197 PMC3727385 19641587

[21] Hershberger KA AS M Hirschey MD. Role of NAD+ and Mitochondrial Sirtuins in Cardiac and Renal Diseases. Nat Rev Nephrol (2017) 13:213–25. doi: 10.1038/nrneph.2017.5 PMC5508210 28163307

[22] Wang M Lin H. Understanding the Function of Mammalian Sirtuins and Protein Lysine Acylation. Annu Rev Biochem (2021) 90:245–85. doi: 10.1146/annurev-biochem-082520-125411 33848425

[23] Xie N Zhang L Gao W Huang C Huber PE Zhou X. NAD+ Metabolism: Pathophysiologic Mechanisms and Therapeutic Potential. Signal Transduct Target Ther (2020) 5:227. doi: 10.1038/s41392-020-00311-7 33028824 PMC7539288

[24] Kitada M Ogura Y Monno I Koya D. Sirtuins and Type 2 Diabetes: Role in Inflammation, Oxidative Stress, and Mitochondrial Function. Front Endocrinol (Lausanne) (2019) 10:187. doi: 10.3389/fendo.2019.00187 30972029 PMC6445872

[25] Zhu S Dong Z Ke X Hou J Zhao E Zhang K. The Roles of Sirtuins Family in Cell Metabolism During Tumor Development. Semin Cancer Biol (2019) 57:59–71. doi: 10.1016/j.semcancer.2018.11.003 30453040

[26] Zhou S Tang X Chen H-Z. Sirtuins and Insulin Resistance. Front Endocrinol (Lausanne) (2018) 9:748. doi: 10.3389/fendo.2018.00748 30574122 PMC6291425

[27] Tang X Ma H Han L Zheng W Lu Y-B Chen X-F. SIRT1 Deacetylates the Cardiac Transcription Factor Nkx2.5 and Inhibits its Transcriptional Activity. Sci Rep (2016) 6:36576. doi: 10.1038/srep36576 27819261 PMC5098195

[28] Tang X Chen X-F Chen H-Z Liu D-P. Mitochondrial Sirtuins in Cardiometabolic Diseases. Clin Sci (Lond) (2017) 131:2063–78. doi: 10.1042/CS20160685 28739840

[29] Tang X Chen X-F Wang N-Y Wang X-M Liang S-T Zheng W. SIRT2 Acts as a Cardioprotective Deacetylase in Pathological Cardiac Hypertrophy. Circulation (2017) 136:2051–67. doi: 10.1161/CIRCULATIONAHA.117.028728 PMC5698109 28947430

[30] Grootaert MOJ Bennett MR. Sirtuins in Atherosclerosis: Guardians of Healthspan and Therapeutic Targets. Nat Rev Cardiol (2022). doi: 10.1038/s41569-022-00685-x 35354967

[31] Giovannini L Bianchi S. Role of Nutraceutical SIRT1 Modulators in AMPK and mTOR Pathway: Evidence of a Synergistic Effect. Nutrition (2017) 34:82–96. doi: 10.1016/j.nut.2016.09.008 28063518

[32] Zullo A Simone E Grimaldi M Musto V Mancini FP. Sirtuins as Mediator of the Anti-Ageing Effects of Calorie Restriction in Skeletal and Cardiac Muscle. Int J Mol Sci (2018) 19(4):928. doi: 10.3390/ijms19040928 PMC5979282 29561771

[33] Singh CK Chhabra G Ndiaye MA Garcia-Peterson LM Mack NJ Ahmad N. The Role of Sirtuins in Antioxidant and Redox Signaling. Antioxid Redox Signal (2018) 28:643–61. doi: 10.1089/ars.2017.7290 PMC5824489 28891317

[34] Singh P Hanson PS Morris CM. SIRT1 Ameliorates Oxidative Stress Induced Neural Cell Death and is Down-Regulated in Parkinson’s Disease. BMC Neurosci (2017) 18:46. doi: 10.1186/s12868-017-0364-1 28578695 PMC5455114

[35] Rardin MJ He W Nishida Y Newman JC Carrico C Danielson SR. SIRT5 Regulates the Mitochondrial Lysine Succinylome and Metabolic Networks. Cell Metab (2013) 18:920–33. doi: 10.1016/j.cmet.2013.11.013 PMC4105152 24315375

[36] Vazquez BN Thackray JK Simonet NG Kane-Goldsmith N Martinez-Redondo P Nguyen T. SIRT7 Promotes Genome Integrity and Modulates non-Homologous End Joining DNA Repair. EMBO J (2016) 35:1488–503. doi: 10.15252/embj.201593499 PMC4884211 27225932

[37] Imai S. Dissecting Systemic Control of Metabolism and Aging in the NAD World: The Importance of SIRT1 and NAMPT-Mediated NAD Biosynthesis. FEBS Lett (2011) 585:1657–62. doi: 10.1016/j.febslet.2011.04.060 PMC3104082 21550345

[38] Imai S Yoshino J. The Importance of NAMPT/NAD/SIRT1 in the Systemic Regulation of Metabolism and Ageing. Diabetes Obes Metab (2013) 15 Suppl 3:26–33. doi: 10.1111/dom.12171 24003918 PMC3819727

[39] Morigi M Perico L Benigni A. Sirtuins in Renal Health and Disease. J Am Soc Nephrol (2018) 29:1799–809. doi: 10.1681/ASN.2017111218 PMC6050939 29712732

[40] Jung YJ Lee AS Nguyen-Thanh T Kim D Kang KP Lee S. SIRT2 Regulates LPS-Induced Renal Tubular CXCL2 and CCL2 Expression. J Am Soc Nephrol (2015) 26:1549–60. doi: 10.1681/ASN.2014030226 PMC4483578 25349202

[41] Koyama T Kume S Koya D Araki S Isshiki K Chin-Kanasaki M. SIRT3 Attenuates Palmitate-Induced ROS Production and Inflammation in Proximal Tubular Cells. Free Radic Biol Med (2011) 51:1258–67. doi: 10.1016/j.freeradbiomed.2011.05.028 21664458

[42] Chiba T Peasley KD Cargill KR Maringer K v Bharathi SS Mukherjee E. Sirtuin 5 Regulates Proximal Tubule Fatty Acid Oxidation to Protect Against AKI. J Am Soc Nephrol (2019) 30:2384–98. doi: 10.1681/ASN.2019020163 PMC6900790 31575700

[43] Huang W Liu H Zhu S Woodson M Liu R Tilton RG. Sirt6 Deficiency Results in Progression of Glomerular Injury in the Kidney. Aging (2017) 9:1069–83. doi: 10.18632/aging.101214 PMC5391219 28351995

[44] Liu M Liang K Zhen J Zhou M Wang X Wang Z. Sirt6 Deficiency Exacerbates Podocyte Injury and Proteinuria Through Targeting Notch Signaling. Nat Commun (2017) 8:413. doi: 10.1038/s41467-017-00498-4 28871079 PMC5583183

[45] Cai J Liu Z Huang X Shu S Hu X Zheng M. The Deacetylase Sirtuin 6 Protects Against Kidney Fibrosis by Epigenetically Blocking β-Catenin Target Gene Expression. Kidney Int (2020) 97:106–18. doi: 10.1016/j.kint.2019.08.028 31787254

[46] Miyasato Y Yoshizawa T Sato Y Nakagawa T Miyasato Y Kakizoe Y. Sirtuin 7 Deficiency Ameliorates Cisplatin-Induced Acute Kidney Injury Through Regulation of the Inflammatory Response. Sci Rep (2018) 8:5927. doi: 10.1038/s41598-018-24257-7 29651144 PMC5897539

[47] Bian C Gao J Wang Y Li J Luan Z Lu H. Association of SIRT6 Circulating Levels With Urinary and Glycometabolic Markers in Pre-Diabetes and Diabetes. Acta Diabetol (2021) 58:1551–62. doi: 10.1007/s00592-021-01759-x 34148121

[48] Lenzen S. The Mechanisms of Alloxan- and Streptozotocin-Induced Diabetes. Diabetologia (2008) 51:216–26. doi: 10.1007/s00125-007-0886-7 18087688

[49] Goyal SN Reddy NM Patil KR Nakhate KT Ojha S Patil CR. Challenges and Issues With Streptozotocin-Induced Diabetes - A Clinically Relevant Animal Model to Understand the Diabetes Pathogenesis and Evaluate Therapeutics. Chem Biol Interact (2016) 244:49–63. doi: 10.1016/j.cbi.2015.11.032 26656244

[50] Gheibi S Kashfi K Ghasemi A. A Practical Guide for Induction of Type-2 Diabetes in Rat: Incorporating a High-Fat Diet and Streptozotocin. Biomed Pharmacother = Biomed pharmacotherapie (2017) 95:605–13. doi: 10.1016/j.biopha.2017.08.098 28881291

[51] Kume S Haneda M Kanasaki K Sugimoto T Araki S Isshiki K. SIRT1 Inhibits Transforming Growth Factor Beta-Induced Apoptosis in Glomerular Mesangial Cells via Smad7 Deacetylation. J Biol Chem (2007) 282:151–8. doi: 10.1074/jbc.M605904200 17098745

[52] Tikoo K Singh K Kabra D Sharma V Gaikwad A. Change in Histone H3 Phosphorylation, MAP Kinase P38, SIR 2 and P53 Expression by Resveratrol in Preventing Streptozotocin Induced Type I Diabetic Nephropathy. Free Radic Res (2008) 42:397–404. doi: 10.1080/10715760801998646 18404539

[53] Hasegawa K Wakino S Yoshioka K Tatematsu S Hara Y Minakuchi H. Sirt1 Protects Against Oxidative Stress-Induced Renal Tubular Cell Apoptosis by the Bidirectional Regulation of Catalase Expression. Biochem Biophys Res Commun (2008) 372:51–6. doi: 10.1016/j.bbrc.2008.04.176 18485895

[54] Kitada M Kume S Imaizumi N Koya D. Resveratrol Improves Oxidative Stress and Protects Against Diabetic Nephropathy Through Normalization of Mn-SOD Dysfunction in AMPK/SIRT1-Independent Pathway. Diabetes (2011) 60:634–43. doi: 10.2337/db10-0386 PMC3028365 21270273

[55] Chuang PY Dai Y Liu R He H Kretzler M Jim B. Alteration of Forkhead Box O (Foxo4) Acetylation Mediates Apoptosis of Podocytes in Diabetes Mellitus. PLoS One (2011) 6:e23566. doi: 10.1371/journal.pone.0023566 21858169 PMC3157434

[56] Kitada M Takeda A Nagai T Ito H Kanasaki K Koya D. Dietary Restriction Ameliorates Diabetic Nephropathy Through Anti-Inflammatory Effects and Regulation of the Autophagy via Restoration of Sirt1 in Diabetic Wistar Fatty (Fa/Fa) Rats: A Model of Type 2 Diabetes. Exp Diabetes Res (2011) 2011:908185. doi: 10.1155/2011/908185 21949662 PMC3178150

[57] Yuan Y Huang S Wang W Wang Y Zhang P Zhu C. Activation of Peroxisome Proliferator-Activated Receptor-γ Coactivator 1α Ameliorates Mitochondrial Dysfunction and Protects Podocytes From Aldosterone-Induced Injury. Kidney Int (2012) 82:771–89. doi: 10.1038/ki.2012.188 22648295

[58] Wu L Zhang Y Ma X Zhang N Qin G. The Effect of Resveratrol on FoxO1 Expression in Kidneys of Diabetic Nephropathy Rats. Mol Biol Rep (2012) 39:9085–93. doi: 10.1007/s11033-012-1780-z 22733486

[59] Kim MY Lim JH Youn HH Hong YA Yang KS Park HS. Resveratrol Prevents Renal Lipotoxicity and Inhibits Mesangial Cell Glucotoxicity in a Manner Dependent on the AMPK-SIRT1-Pgc1α Axis in Db/Db Mice. Diabetologia (2013) 56:204–17. doi: 10.1007/s00125-012-2747-2 23090186

[60] Huang K Huang J Xie X Wang S Chen C Shen X. Sirt1 Resists Advanced Glycation End Products-Induced Expressions of Fibronectin and TGF-β1 by Activating the Nrf2/ARE Pathway in Glomerular Mesangial Cells. Free Radic Biol Med (2013) 65:528–40. doi: 10.1016/j.freeradbiomed.2013.07.029 23891678

[61] Hasegawa K Wakino S Simic P Sakamaki Y Minakuchi H Fujimura K. Renal Tubular Sirt1 Attenuates Diabetic Albuminuria by Epigenetically Suppressing Claudin-1 Overexpression in Podocytes. Nat Med (2013) 19:1496–504. doi: 10.1038/nm.3363 PMC4041199 24141423

[62] Wen D Huang X Zhang M Zhang L Chen J Gu Y. Resveratrol Attenuates Diabetic Nephropathy via Modulating Angiogenesis. PLoS One (2013) 8:e82336. doi: 10.1371/journal.pone.0082336 24312656 PMC3849393

[63] Liu R Zhong Y Li X Chen H Jim B Zhou M-M. Role of Transcription Factor Acetylation in Diabetic Kidney Disease. Diabetes (2014) 63:2440–53. doi: 10.2337/db13-1810 PMC4066331 24608443

[64] Tikoo K Lodea S Karpe PA Kumar S. Calorie Restriction Mimicking Effects of Roflumilast Prevents Diabetic Nephropathy. Biochem Biophys Res Commun (2014) 450:1581–6. doi: 10.1016/j.bbrc.2014.07.039 25035926

[65] Papadimitriou A Silva KC Peixoto EB Borges CM Lopes de Faria JM Lopes de Faria JB. Theobromine Increases NAD+/Sirt-1 Activity and Protects the Kidney Under Diabetic Conditions. Am J Physiol Renal Physiol (2015) 308(3):F209–25. doi: 10.1152/ajprenal.00252.2014 25411384

[66] Mortuza R Feng B Chakrabarti S. SIRT1 Reduction Causes Renal and Retinal Injury in Diabetes Through Endothelin 1 and Transforming Growth Factor β1. J Cell Mol Med (2015) 19:1857–67. doi: 10.1111/jcmm.12557 PMC4549036 25753689

[67] Shao Y Lv C Wu C Zhou Y Wang Q. Mir-217 Promotes Inflammation and Fibrosis in High Glucose Cultured Rat Glomerular Mesangial Cells via Sirt1/HIF-1α Signaling Pathway. Diabetes Metab Res Rev (2016) 32:534–43. doi: 10.1002/dmrr.2788 26891083

[68] Park HS Lim JH Kim MY Kim Y Hong YA Choi SR. Resveratrol Increases AdipoR1 and AdipoR2 Expression in Type 2 Diabetic Nephropathy. J Transl Med (2016) 14:176. doi: 10.1186/s12967-016-0922-9 27286657 PMC4902973

[69] Hussein MMA Mahfouz MK. Effect of Resveratrol and Rosuvastatin on Experimental Diabetic Nephropathy in Rats. Biomed Pharmacother = Biomed pharmacotherapie (2016) 82:685–92. doi: 10.1016/j.biopha.2016.06.004 27470412

[70] Wang X-L Wu L-Y Zhao L Sun L-N Liu H-Y Liu G. SIRT1 Activator Ameliorates the Renal Tubular Injury Induced by Hyperglycemia In Vivo and In Vitro via Inhibiting Apoptosis. Biomed Pharmacother = Biomed pharmacotherapie (2016) 83:41–50. doi: 10.1016/j.biopha.2016.06.009 27470548

[71] Iskender H Dokumacioglu E Sen TM Ince I Kanbay Y Saral S. The Effect of Hesperidin and Quercetin on Oxidative Stress, NF-κb and SIRT1 Levels in a STZ-Induced Experimental Diabetes Model. Biomed Pharmacother = Biomed pharmacotherapie (2017) 90:500–8. doi: 10.1016/j.biopha.2017.03.102 28395272

[72] Li X Cai W Lee K Liu B Deng Y Chen Y. Puerarin Attenuates Diabetic Kidney Injury Through the Suppression of NOX4 Expression in Podocytes. Sci Rep (2017) 7:14603. doi: 10.1038/s41598-017-14906-8 29097815 PMC5668268

[73] Hong Q Zhang L Das B Li Z Liu B Cai G. Increased Podocyte Sirtuin-1 Function Attenuates Diabetic Kidney Injury. Kidney Int (2018) 93:1330–43. doi: 10.1016/j.kint.2017.12.008 PMC5967974 29477240

[74] Li A Peng R Sun Y Liu H Peng H Zhang Z. LincRNA 1700020I14Rik Alleviates Cell Proliferation and Fibrosis in Diabetic Nephropathy via miR-34a-5p/Sirt1/HIF-1α Signaling. Cell Death Dis (2018) 9:461. doi: 10.1038/s41419-018-0527-8 29700282 PMC5919933

[75] Umino H Hasegawa K Minakuchi H Muraoka H Kawaguchi T Kanda T. High Basolateral Glucose Increases Sodium-Glucose Cotransporter 2 and Reduces Sirtuin-1 in Renal Tubules Through Glucose Transporter-2 Detection. Sci Rep (2018) 8:6791. doi: 10.1038/s41598-018-25054-y 29717156 PMC5931531

[76] Wang Y Zheng Z-J Jia Y-J Yang Y-L Xue Y-M. Role of P53/miR-155-5p/Sirt1 Loop in Renal Tubular Injury of Diabetic Kidney Disease. J Transl Med (2018) 16:146. doi: 10.1186/s12967-018-1486-7 29848325 PMC5975703

[77] Sun Z Ma Y Chen F Wang S Chen B Shi J. miR-133b and miR-199b Knockdown Attenuate TGF-β1-Induced Epithelial to Mesenchymal Transition and Renal Fibrosis by Targeting SIRT1 in Diabetic Nephropathy. Eur J Pharmacol (2018) 837:96–104. doi: 10.1016/j.ejphar.2018.08.022 30125566

[78] Zhang T Chi Y Kang Y Lu H Niu H Liu W. Resveratrol Ameliorates Podocyte Damage in Diabetic Mice via SIRT1/PGC-1α Mediated Attenuation of Mitochondrial Oxidative Stress. J Cell Physiol (2019) 234:5033–43. doi: 10.1002/jcp.27306 30187480

[79] Shi S Lei S Tang C Wang K Xia Z. Melatonin Attenuates Acute Kidney Ischemia/Reperfusion Injury in Diabetic Rats by Activation of the SIRT1/Nrf2/HO-1 Signaling Pathway. Biosci Rep (2019) 39(1):BSR20181614. doi: 10.1042/BSR20181614 30578379 PMC6331666

[80] Xue H Li P Luo Y Wu C Liu Y Qin X. Salidroside Stimulates the Sirt1/PGC-1α Axis and Ameliorates Diabetic Nephropathy in Mice. Phytomedicine (2019) 54:240–7. doi: 10.1016/j.phymed.2018.10.031 30668374

[81] Esmaeili S Motamedrad M Hemmati M Mehrpour O Khorashadizadeh M. Prevention of Kidney Cell Damage in Hyperglycaemia Condition by Adiponectin. Cell Biochem Funct (2019) 37:148–52. doi: 10.1002/cbf.3380 30908696

[82] Cai Y-Y Zhang H-B Fan C-X Zeng Y-M Zou S-Z Wu C-Y. Renoprotective Effects of Brown Adipose Tissue Activation in Diabetic Mice. J Diabetes (2019) 11:958–70. doi: 10.1111/1753-0407.12938 PMC6899899 31020790

[83] Zhou D Zhou M Wang Z Fu Y Jia M Wang X. PGRN Acts as a Novel Regulator of Mitochondrial Homeostasis by Facilitating Mitophagy and Mitochondrial Biogenesis to Prevent Podocyte Injury in Diabetic Nephropathy. Cell Death Dis (2019) 10:524. doi: 10.1038/s41419-019-1754-3 31285425 PMC6614416

[84] Ren H Shao Y Wu C Ma X Lv C Wang Q. Metformin Alleviates Oxidative Stress and Enhances Autophagy in Diabetic Kidney Disease via AMPK/SIRT1-FoxO1 Pathway. Mol Cell Endocrinol (2020) 500:110628. doi: 10.1016/j.mce.2019.110628 31647955

[85] Akhtar S Siragy HM. Pro-Renin Receptor Suppresses Mitochondrial Biogenesis and Function via AMPK/SIRT-1/PGC-1α Pathway in Diabetic Kidney. PLoS One (2019) 14:e0225728. doi: 10.1371/journal.pone.0225728 31800607 PMC6892478

[86] Xu J Liu L-Q Xu L-L Xing Y Ye S. Metformin Alleviates Renal Injury in Diabetic Rats by Inducing Sirt1/FoxO1 Autophagic Signal Axis. Clin Exp Pharmacol Physiol (2020) 47:599–608. doi: 10.1111/1440-1681.13226 31821581

[87] Wang S Yang Y He X Yang L Wang J Xia S. Cdk5-Mediated Phosphorylation of Sirt1 Contributes to Podocyte Mitochondrial Dysfunction in Diabetic Nephropathy. Antioxid Redox Signal (2021) 34:171–90. doi: 10.1089/ars.2020.8038 32660255

[88] Chen J Yang Y Lv Z Shu A Du Q Wang W. Study on the Inhibitive Effect of Catalpol on Diabetic Nephropathy. Life Sci (2020) 257:118120. doi: 10.1016/j.lfs.2020.118120 32693244

[89] Alzahrani S SA Z Said E El-Sherbiny M Ajwah S SY A. Protective Effect of Isoliquiritigenin on Experimental Diabetic Nephropathy in Rats: Impact on Sirt-1/Nfκb Balance and NLRP3 Expression. Int Immunopharmacol (2020) 87:106813. doi: 10.1016/j.intimp.2020.106813 32707499

[90] Li F Chen Y Li Y Huang M Zhao W. Geniposide Alleviates Diabetic Nephropathy of Mice Through AMPK/SIRT1/NF-κb Pathway. Eur J Pharmacol (2020) 886:173449. doi: 10.1016/j.ejphar.2020.173449 32758570

[91] Samadi M Aziz SG-G Naderi R. The Effect of Tropisetron on Oxidative Stress, SIRT1, FOXO3a, and Claudin-1 in the Renal Tissue of STZ-Induced Diabetic Rats. Cell Stress Chaperones (2021) 26:217–27. doi: 10.1007/s12192-020-01170-5 PMC7736377 33047279

[92] Fu Y Sun Y Wang M Hou Y Huang W Zhou D. Elevation of JAML Promotes Diabetic Kidney Disease by Modulating Podocyte Lipid Metabolism. Cell Metab (2020) 32:1052–62.e8. doi: 10.1016/j.cmet.2020.10.019 33186558

[93] Sun H-J Xiong S-P Cao X Cao L Zhu M-Y Wu Z-Y. Polysulfide-Mediated Sulfhydration of SIRT1 Prevents Diabetic Nephropathy by Suppressing Phosphorylation and Acetylation of P65 NF-κb and STAT3. Redox Biol (2021) 38:101813. doi: 10.1016/j.redox.2020.101813 33279869 PMC7718489

[94] Zhu H Fang Z Chen J Yang Y Gan J Luo L. PARP-1 and SIRT-1 are Interacted in Diabetic Nephropathy by Activating AMPK/PGC-1α Signaling Pathway. Diabetes Metab Syndr Obes (2021) 14:355–66. doi: 10.2147/DMSO.S291314 PMC7846827 33531822

[95] Xia X Wang X Wang H Lin Z Shao K Xu J. Ameliorative Effect of White Tea From 50-Year-Old Tree of Camellia Sinensis L. (Theaceae) on Kidney Damage in Diabetic Mice via SIRT1/AMPK Pathway. J Ethnopharmacol (2021) 272:113919. doi: 10.1016/j.jep.2021.113919 33577915

[96] Wang F Nguyen M Qin FX-F Tong Q. SIRT2 Deacetylates FOXO3a in Response to Oxidative Stress and Caloric Restriction. Aging Cell (2007) 6:505–14. doi: 10.1111/j.1474-9726.2007.00304.x 17521387

[97] Zhuo L Fu B Bai X Zhang B Wu L Cui J. NAD Blocks High Glucose Induced Mesangial Hypertrophy via Activation of the Sirtuins-AMPK-mTOR Pathway. Cell Physiol Biochem (2011) 27:681–90. doi: 10.1159/000330077 21691086

[98] Kundu A Richa S Dey P Kim KS Son JY Kim HR. Protective Effect of EX-527 Against High-Fat Diet-Induced Diabetic Nephropathy in Zucker Rats. Toxicol Appl Pharmacol (2020) 390:114899. doi: 10.1016/j.taap.2020.114899 31981641

[99] Jiao X Li Y Zhang T Liu M Chi Y. Role of Sirtuin3 in High Glucose-Induced Apoptosis in Renal Tubular Epithelial Cells. Biochem Biophys Res Commun (2016) 480:387–93. doi: 10.1016/j.bbrc.2016.10.060 27773814

[100] Ogura Y Kitada M Monno I Kanasaki K Watanabe A Koya D. Renal Mitochondrial Oxidative Stress is Enhanced by the Reduction of Sirt3 Activity, in Zucker Diabetic Fatty Rats. Redox Rep (2018) 23:153–9. doi: 10.1080/13510002.2018.1487174 PMC6748695 29897845

[101] Srivastava SP Li J Kitada M Fujita H Yamada Y Goodwin JE. SIRT3 Deficiency Leads to Induction of Abnormal Glycolysis in Diabetic Kidney With Fibrosis. Cell Death Dis (2018) 9:997. doi: 10.1038/s41419-018-1057-0 30250024 PMC6155322

[102] Wang Z Li Y Wang Y Zhao K Chi Y Wang B. Pyrroloquinoline Quinine Protects HK-2 Cells Against High Glucose-Induced Oxidative Stress and Apoptosis Through Sirt3 and PI3K/Akt/FoxO3a Signaling Pathway. Biochem Biophys Res Commun (2019) 508:398–404. doi: 10.1016/j.bbrc.2018.11.140 30502093

[103] Liu Z Liu H Xiao L Liu G Sun L He L. STC-1 Ameliorates Renal Injury in Diabetic Nephropathy by Inhibiting the Expression of BNIP3 Through the AMPK/SIRT3 Pathway. Lab Invest (2019) 99:684–97. doi: 10.1038/s41374-018-0176-7 30683904

[104] Locatelli M Zoja C Zanchi C Corna D Villa S Bolognini S. Manipulating Sirtuin 3 Pathway Ameliorates Renal Damage in Experimental Diabetes. Sci Rep (2020) 10:8418. doi: 10.1038/s41598-020-65423-0 32439965 PMC7242337

[105] Ogura Y Kitada M Xu J Monno I Koya D. CD38 Inhibition by Apigenin Ameliorates Mitochondrial Oxidative Stress Through Restoration of the Intracellular NAD+/NADH Ratio and Sirt3 Activity in Renal Tubular Cells in Diabetic Rats. Aging (2020) 12:11325–36. doi: 10.18632/aging.103410 PMC7343471 32507768

[106] Wongmekiat O Lailerd N Kobroob A Peerapanyasut W. Protective Effects of Purple Rice Husk Against Diabetic Nephropathy by Modulating PGC-1α/SIRT3/SOD2 Signaling and Maintaining Mitochondrial Redox Equilibrium in Rats. Biomolecules (2021) 11(8):1224. doi: 10.3390/biom11081224 34439892 PMC8392712

[107] Shi J-X Wang Q-J Li H Huang Q. SIRT4 Overexpression Protects Against Diabetic Nephropathy by Inhibiting Podocyte Apoptosis. Exp Ther Med (2017) 13:342–8. doi: 10.3892/etm.2016.3938 PMC5245066 28123512

[108] Fan Y Yang Q Yang Y Gao Z Ma Y Zhang L. Sirt6 Suppresses High Glucose-Induced Mitochondrial Dysfunction and Apoptosis in Podocytes Through AMPK Activation. Int J Biol Sci (2019) 15:701–13. doi: 10.7150/ijbs.29323 PMC6367578 30745856

[109] Muraoka H Hasegawa K Sakamaki Y Minakuchi H Kawaguchi T Yasuda I. Role of Nampt-Sirt6 Axis in Renal Proximal Tubules in Extracellular Matrix Deposition in Diabetic Nephropathy. Cell Rep (2019) 27:199–212.e5. doi: 10.1016/j.celrep.2019.03.024 30943401

[110] Ji L Chen Y Wang H Zhang W He L Wu J. Overexpression of Sirt6 Promotes M2 Macrophage Transformation, Alleviating Renal Injury in Diabetic Nephropathy. Int J Oncol (2019) 55:103–15. doi: 10.3892/ijo.2019.4800 PMC6561622 31115579

[111] Wang X Ji T Li X Qu X Bai S. FOXO3a Protects Against Kidney Injury in Type II Diabetic Nephropathy by Promoting Sirt6 Expression and Inhibiting Smad3 Acetylation. Oxid Med Cell Longev (2021) 2021:5565761. doi: 10.1155/2021/5565761 34122724 PMC8172321

[112] Liu J Duan P Xu C Xu D Liu Y Jiang J. CircRNA Circ-ITCH Improves Renal Inflammation and Fibrosis in Streptozotocin-Induced Diabetic Mice by Regulating the miR-33a-5p/SIRT6 Axis. Inflammation Res (2021) 70:835–46. doi: 10.1007/s00011-021-01485-8 34216220

[113] Bian C Zhang R Wang Y Li J Song Y Guo D. Sirtuin 6 Affects Glucose Reabsorption and Gluconeogenesis in Type 1 Diabetes via Foxo1. Mol Cell Endocrinol (2022) 547:111597. doi: 10.1016/j.mce.2022.111597 35157928

[114] Kawano K Hirashima T Mori S Saitoh Y Kurosumi M Natori T. Spontaneous Long-Term Hyperglycemic Rat With Diabetic Complications. Otsuka Long-Evans Tokushima Fatty (OLETF) Strain. Diabetes (1992) 41:1422–8. doi: 10.2337/diab.41.11.1422 1397718

[115] Fruen BR Balog EM Schafer J Nitu FR Thomas DD Cornea RL. Direct Detection of Calmodulin Tuning by Ryanodine Receptor Channel Targets Using a Ca2+-Sensitive Acrylodan-Labeled Calmodulin. Biochemistry (2005) 44:278–84. doi: 10.1021/bi048246u 15628869

[116] Chang J-H Gurley SB. Assessment of Diabetic Nephropathy in the Akita Mouse. Methods Mol Biol (2012) 933:17–29. doi: 10.1007/978-1-62703-068-7_2 22893398

[117] Fujita H Fujishima H Chida S Takahashi K Qi Z Kanetsuna Y. Reduction of Renal Superoxide Dismutase in Progressive Diabetic Nephropathy. J Am Soc Nephrol (2009) 20:1303–13. doi: 10.1681/ASN.2008080844 PMC2689908 19470681

[118] Nayak BK Shanmugasundaram K Friedrichs WE Cavaglierii RC Patel M Barnes J. HIF-1 Mediates Renal Fibrosis in OVE26 Type 1 Diabetic Mice. Diabetes (2016) 65:1387–97. doi: 10.2337/db15-0519 PMC4839204 26908870

[119] Clee SM ST N Attie AD. Genetic and Genomic Studies of the BTBR Ob/Ob Mouse Model of Type 2 Diabetes. Am J Ther (2005) 12:491–8. doi: 10.1097/01.mjt.0000178781.89789.25 16280642

[120] Hudkins KL Pichaiwong W Wietecha T Kowalewska J MC B MW S. BTBR Ob/Ob Mutant Mice Model Progressive Diabetic Nephropathy. J Am Soc Nephrol (2010) 21:1533–42. doi: 10.1681/ASN.2009121290 PMC3013527 20634301

[121] Nakagawa T Sato W Glushakova O Heinig M Clarke T Campbell-Thompson M. Diabetic Endothelial Nitric Oxide Synthase Knockout Mice Develop Advanced Diabetic Nephropathy. J Am Soc Nephrol (2007) 18:539–50. doi: 10.1681/ASN.2006050459 17202420

[122] Zhao HJ Wang S Cheng H Zhang M Takahashi T AB F. Endothelial Nitric Oxide Synthase Deficiency Produces Accelerated Nephropathy in Diabetic Mice. J Am Soc Nephrol (2006) 17:2664–9. doi: 10.1681/ASN.2006070798 PMC4618687 16971655

[123] Salami M Salami R Mafi A Aarabi M-H Vakili O Asemi Z. Therapeutic Potential of Resveratrol in Diabetic Nephropathy According to Molecular Signaling. Curr Mol Pharmacol (2021). doi: 10.2174/1874467215666211217122523 34923951

[124] Shankar S Singh G Srivastava RK. Chemoprevention by Resveratrol: Molecular Mechanisms and Therapeutic Potential. Front Biosci (2007) 12:4839–54. doi: 10.2741/2432 17569614

[125] Li K-X Ji M-J Sun H-J. An Updated Pharmacological Insight of Resveratrol in the Treatment of Diabetic Nephropathy. Gene (2021) 780:145532. doi: 10.1016/j.gene.2021.145532 33631244

[126] Movahed A Raj P Nabipour I Mahmoodi M Ostovar A Kalantarhormozi M. Efficacy and Safety of Resveratrol in Type 1 Diabetes Patients: A Two-Month Preliminary Exploratory Trial. Nutrients (2020) 12(1):161. doi: 10.3390/nu12010161 PMC7019753 31935938

[127] Maeda S Koya D Araki S-I Babazono T Umezono T Toyoda M. Association Between Single Nucleotide Polymorphisms Within Genes Encoding Sirtuin Families and Diabetic Nephropathy in Japanese Subjects With Type 2 Diabetes. Clin Exp Nephrol (2011) 15:381–90. doi: 10.1007/s10157-011-0418-0 PMC3110272 21331741

[128] Zhao Y Wei J Hou X Liu H Guo F Zhou Y. SIRT1 Rs10823108 and FOXO1 Rs17446614 Responsible for Genetic Susceptibility to Diabetic Nephropathy. Sci Rep (2017) 7:10285. doi: 10.1038/s41598-017-10612-7 28860538 PMC5579017

[129] Ren H Shao Y Wu C Lv C Zhou Y Wang Q. VASH-1 Regulates Oxidative Stress and Fibrosis in Diabetic Kidney Disease via SIRT1/HIF1α and Tgfβ1/Smad3 Signaling Pathways. Front Mol Biosci (2020) 7:137. doi: 10.3389/fmolb.2020.00137 32754616 PMC7365843

[130] Lenoir O Jasiek M Hénique C Guyonnet L Hartleben B Bork T. Endothelial Cell and Podocyte Autophagy Synergistically Protect From Diabetes-Induced Glomerulosclerosis. Autophagy (2015) 11:1130–45. doi: 10.1080/15548627.2015.1049799 PMC4590611 26039325

[131] DeFronzo RA Norton L Abdul-Ghani M. Renal, Metabolic and Cardiovascular Considerations of SGLT2 Inhibition. Nat Rev Nephrol (2017) 13:11–26. doi: 10.1038/nrneph.2016.170 27941935

[132] Vallon V Thomson SC. Targeting Renal Glucose Reabsorption to Treat Hyperglycaemia: The Pleiotropic Effects of SGLT2 Inhibition. Diabetologia (2017) 60:215–25. doi: 10.1007/s00125-016-4157-3 PMC5884445 27878313

[133] Vallon V KA P Cunard R Schroth J Whaley J SC T. SGLT2 Mediates Glucose Reabsorption in the Early Proximal Tubule. J Am Soc Nephrol (2011) 22:104–12. doi: 10.1681/ASN.2010030246 PMC3014039 20616166

[134] Han J Pang X Shi X Zhang Y Peng Z Xing Y. Ginkgo Biloba Extract EGB761 Ameliorates the Extracellular Matrix Accumulation and Mesenchymal Transformation of Renal Tubules in Diabetic Kidney Disease by Inhibiting Endoplasmic Reticulum Stress. BioMed Res Int (2021) 2021:6657206. doi: 10.1155/2021/6657206 33860049 PMC8009711

[135] Lovisa S Fletcher-Sananikone E Sugimoto H Hensel J Lahiri S Hertig A. Endothelial-To-Mesenchymal Transition Compromises Vascular Integrity to Induce Myc-Mediated Metabolic Reprogramming in Kidney Fibrosis. Sci Signal (2020) 13(635):eaaz2597. doi: 10.1126/scisignal.aaz2597 32518142 PMC7790440

[136] Hogg SJ Beavis PA Dawson MA Johnstone RW. Targeting the Epigenetic Regulation of Antitumour Immunity. Nat Rev Drug Discovery (2020) 19:776–800. doi: 10.1038/s41573-020-0077-5 32929243

[137] Kato M Natarajan R. Epigenetics and Epigenomics in Diabetic Kidney Disease and Metabolic Memory. Nat Rev Nephrol (2019) 15:327–45. doi: 10.1038/s41581-019-0135-6 PMC6889804 30894700

[138] Gong F Miller KM. Histone Methylation and the DNA Damage Response. Mutat Res Rev Mutat Res (2019) 780:37–47. doi: 10.1016/j.mrrev.2017.09.003 31395347 PMC6690396

[139] Lu H-C Dai W-N He L-Y. Epigenetic Histone Modifications in the Pathogenesis of Diabetic Kidney Disease. Diabetes Metab Syndr Obes (2021) 14:329–44. doi: 10.2147/DMSO.S288500 PMC7837569 33519221

[140] Zhong Y Lee K He JC. SIRT1 Is a Potential Drug Target for Treatment of Diabetic Kidney Disease. Front Endocrinol (Lausanne) (2018) 9:624. doi: 10.3389/fendo.2018.00624 30386303 PMC6199382

[141] Cantó C KJ M Auwerx J. NAD(+) Metabolism and the Control of Energy Homeostasis: A Balancing Act Between Mitochondria and the Nucleus. Cell Metab (2015) 22:31–53. doi: 10.1016/j.cmet.2015.05.023 26118927 PMC4487780

[142] Kitada M Kume S Takeda-Watanabe A Kanasaki K Koya D. Sirtuins and Renal Diseases: Relationship With Aging and Diabetic Nephropathy. Clin Sci (Lond) (2013) 124:153–64. doi: 10.1042/CS20120190 PMC3466784 23075334

[143] Kong L Wu H Zhou W Luo M Tan Y Miao L. Sirtuin 1: A Target for Kidney Diseases. Mol Med (2015) 21:87–97. doi: 10.2119/molmed.2014.00211 25587857 PMC4461580

[144] Hong SJ Dawson TM Dawson VL. Nuclear and Mitochondrial Conversations in Cell Death: PARP-1 and AIF Signaling. Trends Pharmacol Sci (2004) 25:259–64. doi: 10.1016/j.tips.2004.03.005 15120492

[145] Lv T Lu Y Liu Y Feng H Li C Sheng W. General Control of Amino Acid Synthesis 5-Like 1-Mediated Acetylation of Manganese Superoxide Dismutase Regulates Oxidative Stress in Diabetic Kidney Disease. Oxid Med Cell Longev (2021) 2021:6691226. doi: 10.1155/2021/6691226 33680286 PMC7906818

[146] Yu L Liu Y Wu Y Liu Q Feng J Gu X. Smad3/Nox4-Mediated Mitochondrial Dysfunction Plays a Crucial Role in Puromycin Aminonucleoside-Induced Podocyte Damage. Cell Signal (2014) 26:2979–91. doi: 10.1016/j.cellsig.2014.08.030 25229402

[147] Bian C Zhang H Gao J Wang Y Li J Guo D. SIRT6 Regulates SREBP1c-Induced Glucolipid Metabolism in Liver and Pancreas via the Ampkα-Mtorc1 Pathway. Lab Invest (2022) 102(5):474–84. doi: 10.1038/s41374-021-00715-1 34923569

